# The helicase DinG responds to stress due to DNA double strand breaks

**DOI:** 10.1371/journal.pone.0187900

**Published:** 2017-11-09

**Authors:** Stephan A. Frye, Getachew Tesfaye Beyene, Amine Namouchi, Marta Gómez-Muñoz, Håvard Homberset, Shewit Kalayou, Tahira Riaz, Tone Tønjum, Seetha V. Balasingham

**Affiliations:** 1 Department of Microbiology, Oslo University Hospital, Oslo, Norway; 2 Department of Microbiology, University of Oslo, Oslo, Norway; Tulane University Health Sciences Center, UNITED STATES

## Abstract

*Neisseria meningitidis* (Nm) is a Gram-negative nasopharyngeal commensal that can cause septicaemia and meningitis. The neisserial DNA damage-inducible protein DinG is a helicase related to the mammalian helicases XPD and FANCJ. These helicases belong to superfamily 2, are ATP dependent and exert 5′ → 3′ directionality. To better understand the role of DinG in neisserial genome maintenance, the Nm DinG (DinG_Nm_) enzymatic activities were assessed *in vitro* and phenotypical characterization of a *dinG* null mutant (NmΔ*dinG*) was performed. Like its homologues, DinG_Nm_ possesses 5′ → 3′ directionality and prefers DNA substrates containing a 5′-overhang. ATPase activity of DinG_Nm_ is strictly DNA-dependent and DNA unwinding activity requires nucleoside triphosphate and divalent metal cations. DinG_Nm_ directly binds SSB_Nm_ with a K_d_ of 313 nM. Genotoxic stress analysis demonstrated that NmΔ*dinG* was more sensitive to double-strand DNA breaks (DSB) induced by mitomycin C (MMC) than the Nm wildtype, defining the role of neisserial DinG in DSB repair. Notably, when NmΔ*dinG* cells grown under MMC stress assessed by quantitative mass spectrometry, 134 proteins were shown to be differentially abundant (DA) compared to unstressed NmΔ*dinG* cells. Among the DNA replication, repair and recombination proteins affected, polymerase III subunits and recombinational repair proteins RuvA, RuvB, RecB and RecD were significantly down regulated while TopA and SSB were upregulated under stress condition. Most of the other DA proteins detected are involved in metabolic functions. The present study shows that the helicase DinG is probably involved in regulating metabolic pathways as well as in genome maintenance.

## Introduction

*Neisseria meningitidis* (Nm) is a Gram-negative bacterium that frequently colonizes the human nasopharynx in small children and adolescents. In the lack of bactericidal antibodies, Nm can enter the bloodstream and cross the blood-brain barrier leading to septicaemia and meningitis, respectively [1]. We are interested in how Nm cells survive on the oral mucosal surface, in the bloodstream and the meninges, where it is exposed to the host defence, including DNA damaging reactive oxygen and nitrogen species [2]. Therefore, we postulate that DNA repair pathways that promote genome stability play important roles in the survival of Nm under genotoxic stress.

Many proteins involved in DNA repair pathways have multiple overlapping functions and crosstalk between these pathways has been identified [3]. However, the functions of many DNA repair proteins are still not clearly discerned. In this study, we aimed to understand the functional role of the neisserial helicase DinG in the genome maintenance of Nm under genotoxic stress.

Helicases are molecular motor proteins that unwind double stranded nucleic acids using the energy provided by ATP hydrolysis. By doing so, helicases facilitate various aspects of nucleic acid metabolism such as replication, repair, recombination, transcription, translation and splicing of RNA transcripts [4]. Helicases are classified into six superfamilies (SF1-6) based on the sequence identity among the conserved helicase motifs [5]. Helicases belonging to the SF1 and SF2 categories share a catalytic core with high structural similarity, but even within each SF the different enzymes exert distinct functions on diverse nucleic acid substrates [6].

The *Escherichia coli* DNA damage-inducible protein DinG (DinG_Ec_) belongs to the SF2 helicases which translocates on single-stranded DNA (ssDNA) in the 5′→3′ direction [7]. A recent report suggested that DinG_Ec_ is involved in the dissolution of R-loops during replication restart following the collision of replication forks with the transcription unit [8]. It was also reported to be able to resolve intermolecular but not intramolecular G4 DNA [9]. DinG_Ec_ is a structure-specific enzyme, related to human xeroderma pigmentosum group D (XPD), FANCJ, also known as BACH1, as well as to *Saccharomyces cerevisiae* Rad3 and Chl1, and *Schizosaccharomyces pombe* Rad15 [10]. XPD is a subunit of TFIIH, a large multiprotein complex that plays a dual role in transcription initiation and nucleotide excision repair [11]. Mutations in the human XPD helicase gene are found in patients with three inherited diseases: xeroderma pigmentosum (XP), Cockayne syndrome (CS) and trichothiodystrophy (TTD) [12]. Mutations in the gene encoding FANCJ predispose individuals to breast cancer, suggesting a tumour suppressor role for FANCJ [13–15]. The role of the helicase DinG in bacteria is not as well defined, and deletion or overexpression of *dinG* in *E*. *coli* results in poorly discernible phenotypes [7].

In order to define the biological role of DinG helicase in neisserial genome maintenance and bacterial survival, we analysed an Nm wildtype and a *dinG* null mutant, NmΔ*dinG*. The fitness of these Nm strains under various forms of genotoxic stress was assessed and protein expression levels were compared. The gene encoding the Nm helicase DinG (DinG_Nm_) was cloned and overexpressed, the recombinant DinG_Nm_ protein was purified to homogeneity and its enzymatic activities were characterized. Taken together, the results show that DinG_Nm_ responds to stress due to DNA double strand breaks and possibly is involved in metabolic pathway regulation.

## Results

### DinG_Nm_ is a conserved protein among *N*. *meningitidis* isolates

**Fig 1:**
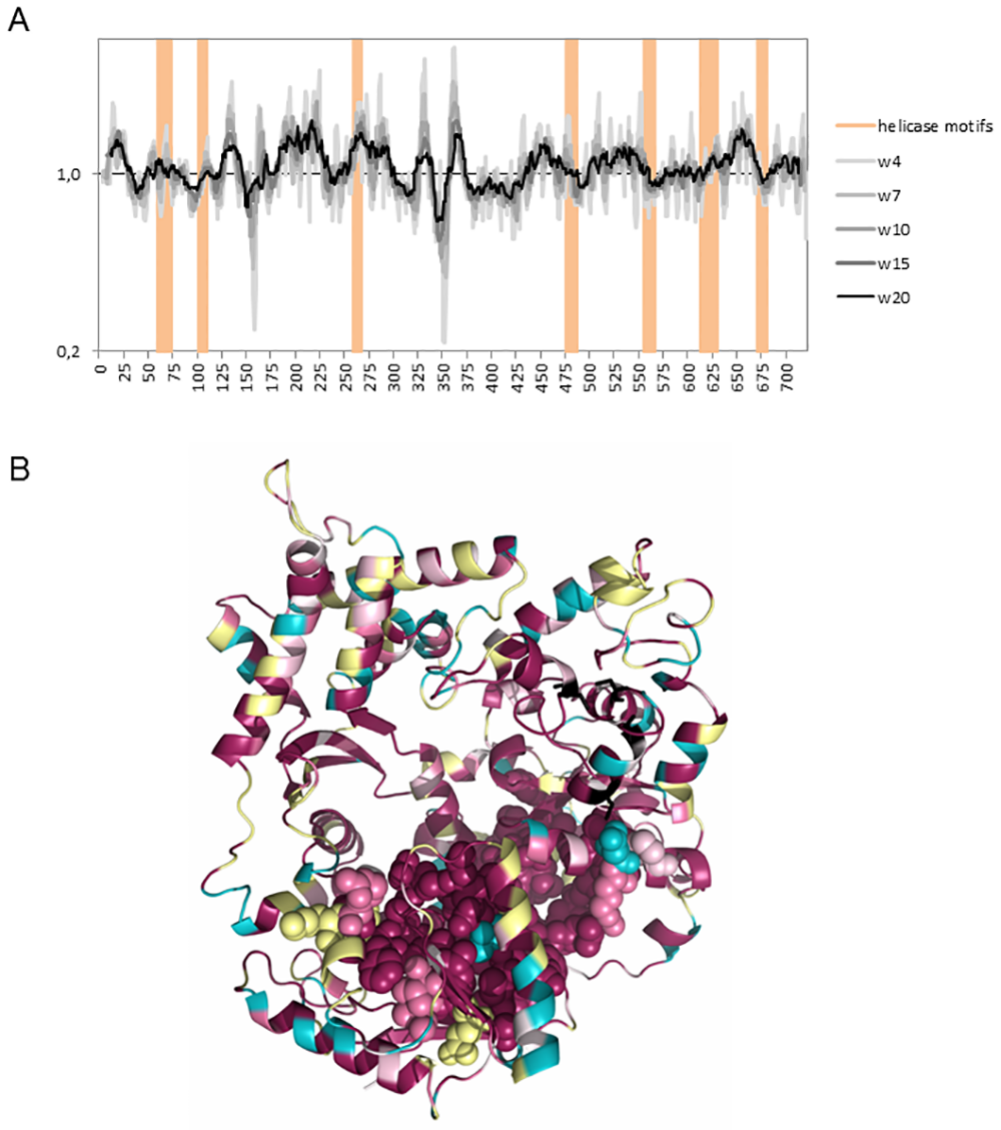
Computational analysis of DinG_Nm_. A) Plotting of the similarity of DinG_Nm_ sequences from PubMLST with sliding window sizes (w) ranging from 4 to 20 aa. Data were generated with plotcon (EMBOSS) and the average similarity for the whole sequence was set to 1. The helicase motive I, Ia, II, III, IV, V, and VI are under laid in orange colour. B) Similarity of the DinG_Nm_ sequences taken from PubMLST plotted onto the predicted structure of DinG_Nm_ from MC58 using default Consurf colouring with cysteins shown in black. The helicase motifs are shown as spheres and the rest of the protein is shown as cartoon.

The 467 *dinG* variants available in PubMLST (PubMLST ID: **NEIS0293**) exhibit only minor variation, with the seven helicase motifs showing strong conservation (Fig 1). Among the 66 amino acid positions exhibiting variations located in the helicase motifs, only 9 are parsimony-informative sites (S1 Fig). According to SNAP2 predictions, all variants at these nine sites impose a neutral functional effect. The sequence of DinG_Nm_ from strain MC58 readily aligns to the sequence of DinG_Ec_ identifying amino acid K72 in DinG_Nm_ as equivalent to the amino acid K60 required for ATPase activity in DinG_Ec_ [7]. The four conserved DinG cysteine residues numbered Cys-120, -194, -199 and -205 in *E*. *coli* [10], correspond to Cys-133, -209, -214 and -220 in Nm.

While the *dinG* from Nm strain MC58 (PubMLST ID: **NEIS0293:3**) does not contain a canonical DNA uptake sequence (DUS), two variant forms of DUS were detected in neisserial *dinG* as one sequence position contains a mucDUS (nt 1023–1032 in **NEIS0293:3**) and another position contains a simDUS (nt 1506–1515 in **NEIS0293:3**) [16].

### DinG_Nm_ is an iron containing DNA dependent ATPase and the DinG_NmK72A_ mutant protein is inactive

**Fig 2:**
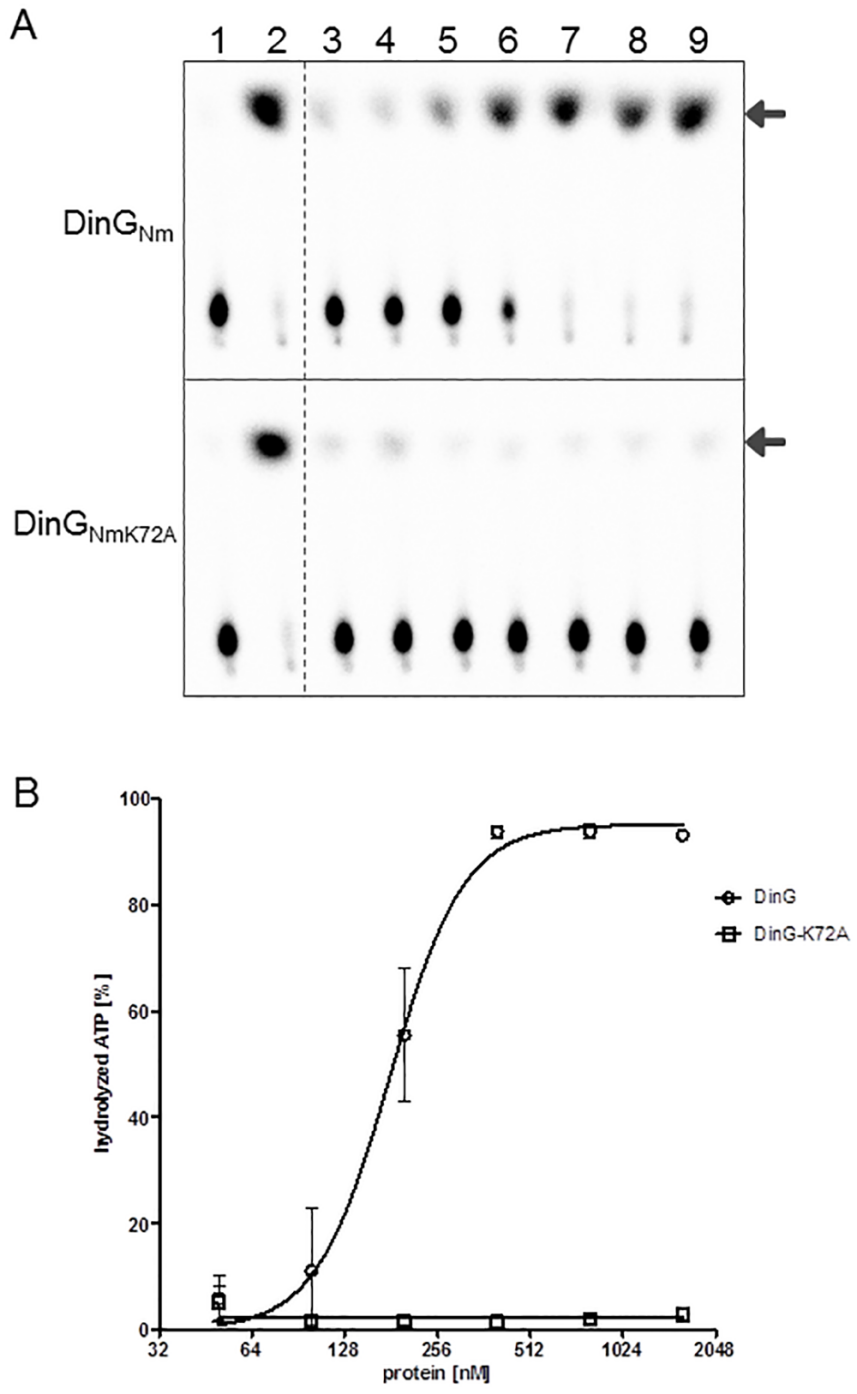
DNA dependent ATPase activity of DinG. A) Representative chromatograms of ATPase activity of DinG_Nm_ (upper panel) and the mutant DinG_NmK72A_ (lower panel). Lanes: 1) 400 nM DinG protein; 2) 90 nM *E*. *coli* UvrD; 3–9) 0, 50, 100, 200, 400, 800, and 1600 nM DinG protein. Lane 2–9 contained 200 nM dT_100_ as DNA cofactor. The released phosphate is indicated by the arrows. B) Graph showing ATPase activities of DinG_Nm_ and DinG_Nm_K72A in the presence of DNA as cofactor. The standard deviations from three independent experiments are indicated by bars.

The recombinant DinG_Nm_ and DinG_NmK72A_ (an ATPase inactive mutant) proteins were purified to homogeneity. We observed that both proteins exhibited a yellow-brown colour which is an attribute for Fe-S proteins such as XPD [17] and AddAB [18]. Using an iron chelation assay, we determined that DinG_Nm_ contained four iron molecules per one protein molecule. The ATP hydrolysis activity of both proteins was tested in the presence of poly dT_100_ as single stranded (ss) DNA cofactor. The ATP hydrolysis activity increased with increasing DinG_Nm_ protein concentration, and nearly 100% of the ATP was hydrolysed in the presence of 400 nM DinG_Nm_ under the conditions used (Fig 2). In the absence of ssDNA as a cofactor, DinG_Nm_ did not show any ATPase activity, indicating that the enzyme depends on DNA for this activity (Fig 2A, lane 1). The mutant protein DinG_NmK72A_ had no ATPase activity under any condition (Fig 2).

### DinG_Nm_ and DinG_NmK72A_ bind to DNA

**Fig 3:**
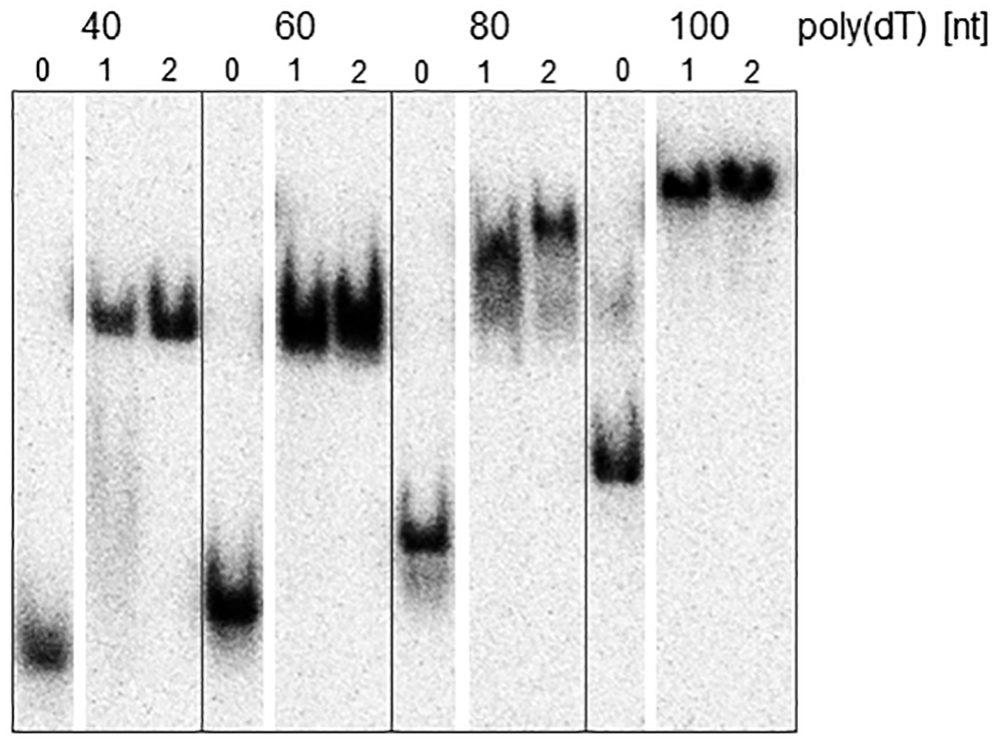
DinG_Nm_ binding to ssDNA. Representative gel images of DNA binding assays containing 800 nM DinG_Nm_ (lane 1), or 800 nM DinG_NmK72A_ (lane 2) and 100 pM homopolymeric nucleotides of given size. Lane 0 contains the control reaction without proteins.

The binding affinities of DinG_Nm_ and the DinG_NmK72A_ were examined in the presence of ssDNA substrates of variable length. Both proteins bound ssDNA with similar affinity with an ssDNA of 40 nt length being sufficient for a complete shift under the conditions tested (Fig 3).

**Fig 4:**
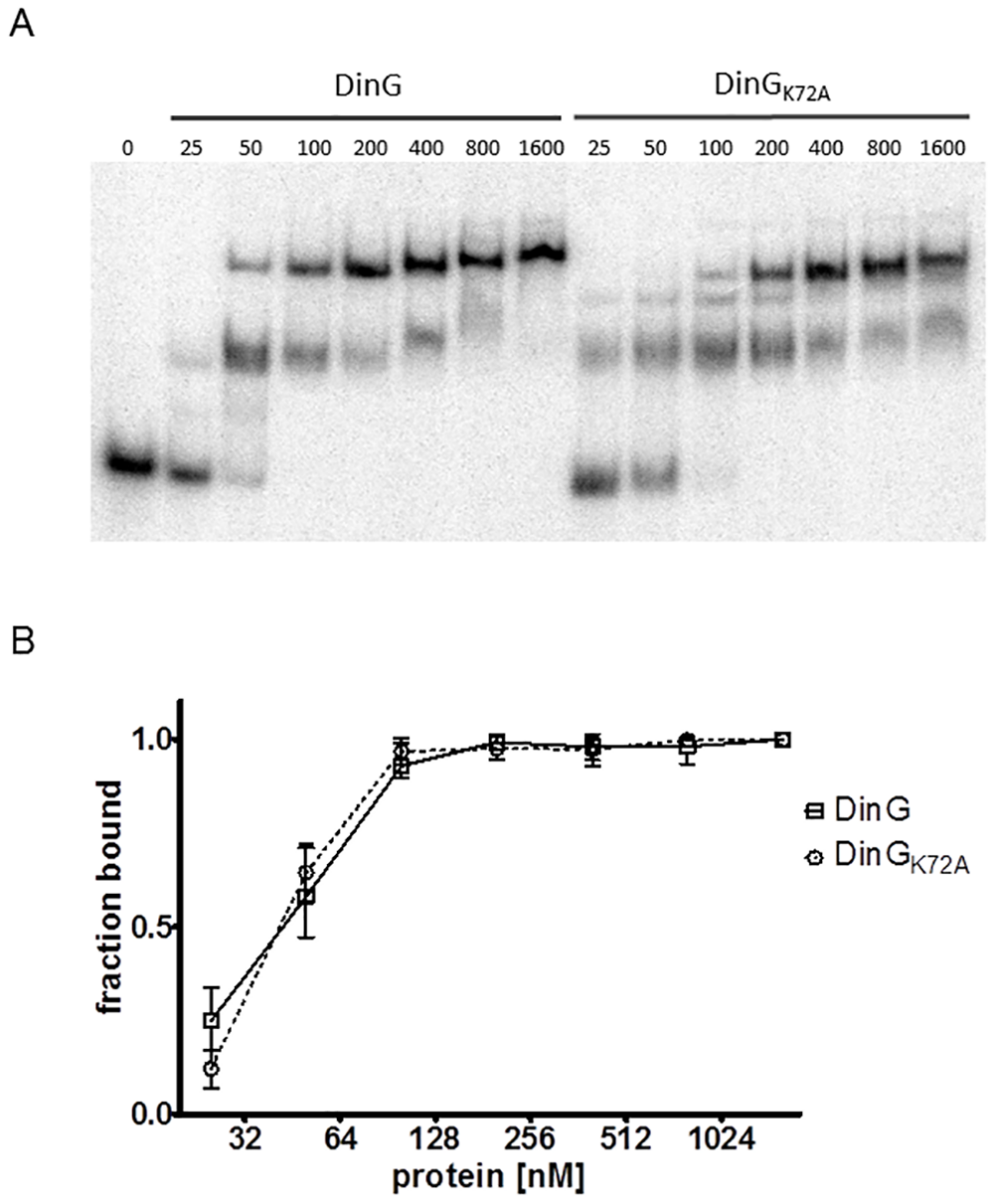
Protein concentration dependent DNA binding. A) DNA binding assay with DinG_Nm_ and DinG_NmK72A_. The protein concentrations are given in nM. B) Quantitation of gel images from three independent experiments. Values are plotted as a fraction of bound DNA versus the protein concentration.

To further examine the DNA binding affinity of these proteins, a 100 nt oligomer substrate (poly dT_100_) was incubated with increasing concentration of the proteins (Fig 4). The results showed that the ATPase mutant protein, DinG_NmK72A_ retained its DNA binding affinity.

### DinG_Nm_ unwinds DNA in the 5′ → 3′ direction

**Fig 5:**
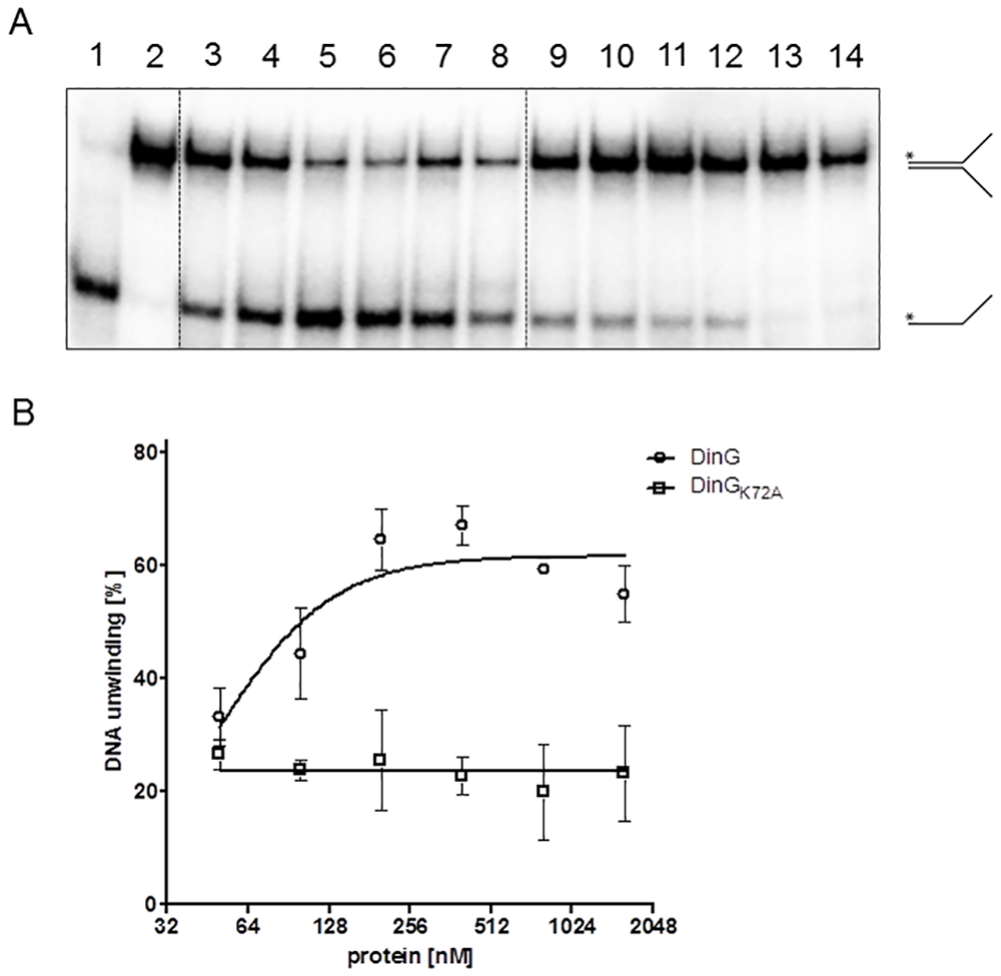
Titration of the DinG_Nm_ DNA unwinding activity. DNA unwinding activity was tested on 1 nM forked DNA substrate (T1+B1 oligo dimer with a 30mer complementary region and 30mer tails) with increasing concentrations of DinG_Nm_ or DinG_NmK72A_. A) A representative gel showing educts and unwinding reaction products, also schematic depicted on the right. Lanes: 1) heat-denatured substrate, 2) no enzyme, 3–8) 50 nM, 100 nM, 200 nM, 400 nM, 800 nM, and 1600 nM DinG_Nm_, respectively, 9–14) 50 nM, 100 nM, 200 nM, 400 nM, 800 nM, and 1600 nM DinG_NmK72A_, respectively. B) Quantitation of the unwinding activity of DinG_Nm_ and DinG_NmK72A_. The average of three independent experiments and standard deviations are shown.

To further investigate the helicase activity of DinG_Nm_, forked DNA substrates were used (S2 Table). Initial studies showed that more ssDNA product was generated from forked DNA substrate (T1+B1) as the concentration of DinG_Nm_ was increased up to 400 nM, while DinG_NmK72A_ failed to unwind the DNA substrate even at higher concentrations (Fig 5).

**Fig 6:**
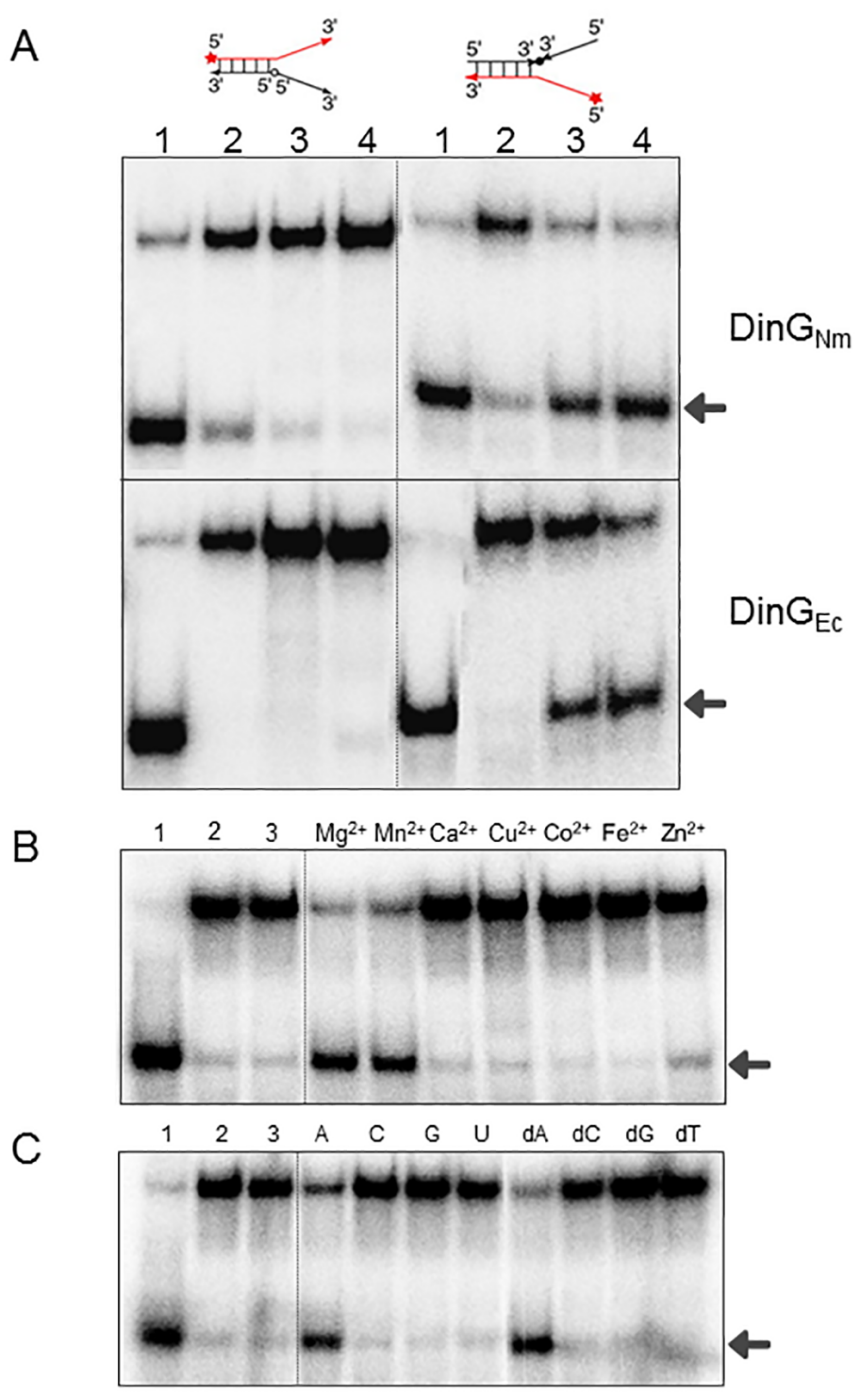
DinG_Nm_ unwinding directionality and cofactor dependency. A) Unwinding activity on modified forked DNA (left: B9+T8-3′-3′, right: T8+B9-5′-5′). Lanes: 1) heat denatured substrate, 2) no protein, 3) 200 nM protein, 4) 400 nM protein. Upper panel: DinG_Nm_, lower panel: DinG_Ec_. B) Metal dependent unwinding. Labelled forked DNA substrate (T1+B1) was incubated with 400 nM DinG_Nm_ in the presence of 2 mM ATP and 2 mM of the metal as indicated on top. 1) heat-denatured DNA substrate, 2) reaction without protein, 3) reaction lacking metal ion. C) Nucleotide dependent unwinding. Labelled forked DNA substrate (T1+B1) was incubated with 400 nM DinG_Nm_ in the presence of 2 mM Mg^2+^ and 2 mM of NTP or dNTP as indicated on top. 1) heat-denatured DNA substrate, 2) reaction without protein, 3) reaction lacking ATP. The products of DNA unwinding are indicated by arrows.

To determine the polarity of the DinG_Nm_-catalysed unwinding, forked DNA substrates with switched polarities were made (T8-3′-3′+ B9 and T8+ B9-5′-5′) such that the unpaired single stranded ends contain either only 5′ or only 3′ ends. As its *E*. *coli* homolog DinG_Ec_, DinG_Nm_ also unwound only the substrate containing open 5′ ends, showing that the unwinding activity has 5′ → 3′ polarity (Fig 6A). The helicase activity of DinG_Nm_ was also shown to be dependent on the presence of Mg^2+^ or Mn^2+^ (Fig 6B). In addition, the unwinding activity of DinG_Nm_ was observed only in the presence of ATP or dATP (Fig 6C), indicating that only the hydrolysis of ATP or dATP can be used by DinG_Nm_ to deliver the energy needed for unwinding.

### DinG_Nm_ directly interacts with SSB_Nm_

**Fig 7:**
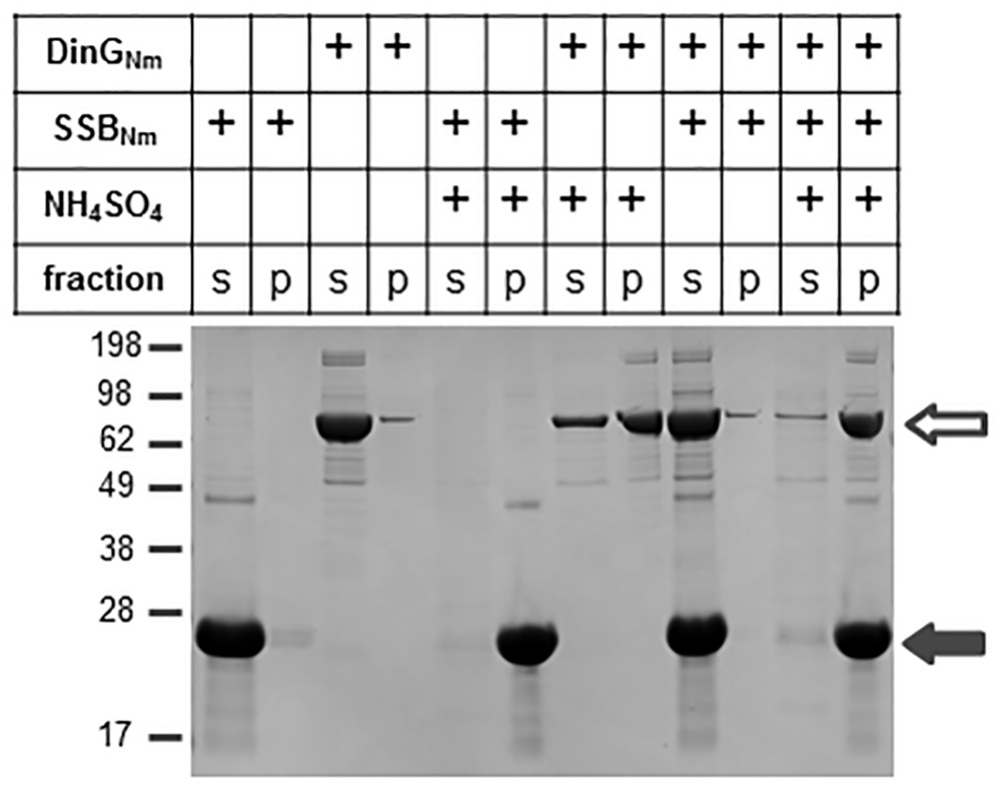
Co-precipitation of DinG_Nm_ and SSB_Nm_. Precipitation of DinG_Nm_ alone and in combination with SSB_Nm_ by ammonium sulphate. An example of a Coomassie blue stained polyacrylamide gel is shown. The open arrow indicates DinG_Nm_ and the filled arrow indicates SSB_Nm_. The supernatant (s) and pellet (p) fractions are shown.

To determine whether DinG_Nm_ interacts with SSB_Nm_, we employed ammonium sulphate co-precipitation and microscale thermophoresis (MST). The precipitation indicated that SSB_Nm_ readily precipitates at 150 g/l ammonium sulphate while only about ½ of DinG_Nm_ precipitates under this conditions (Fig 7).

**Fig 8:**
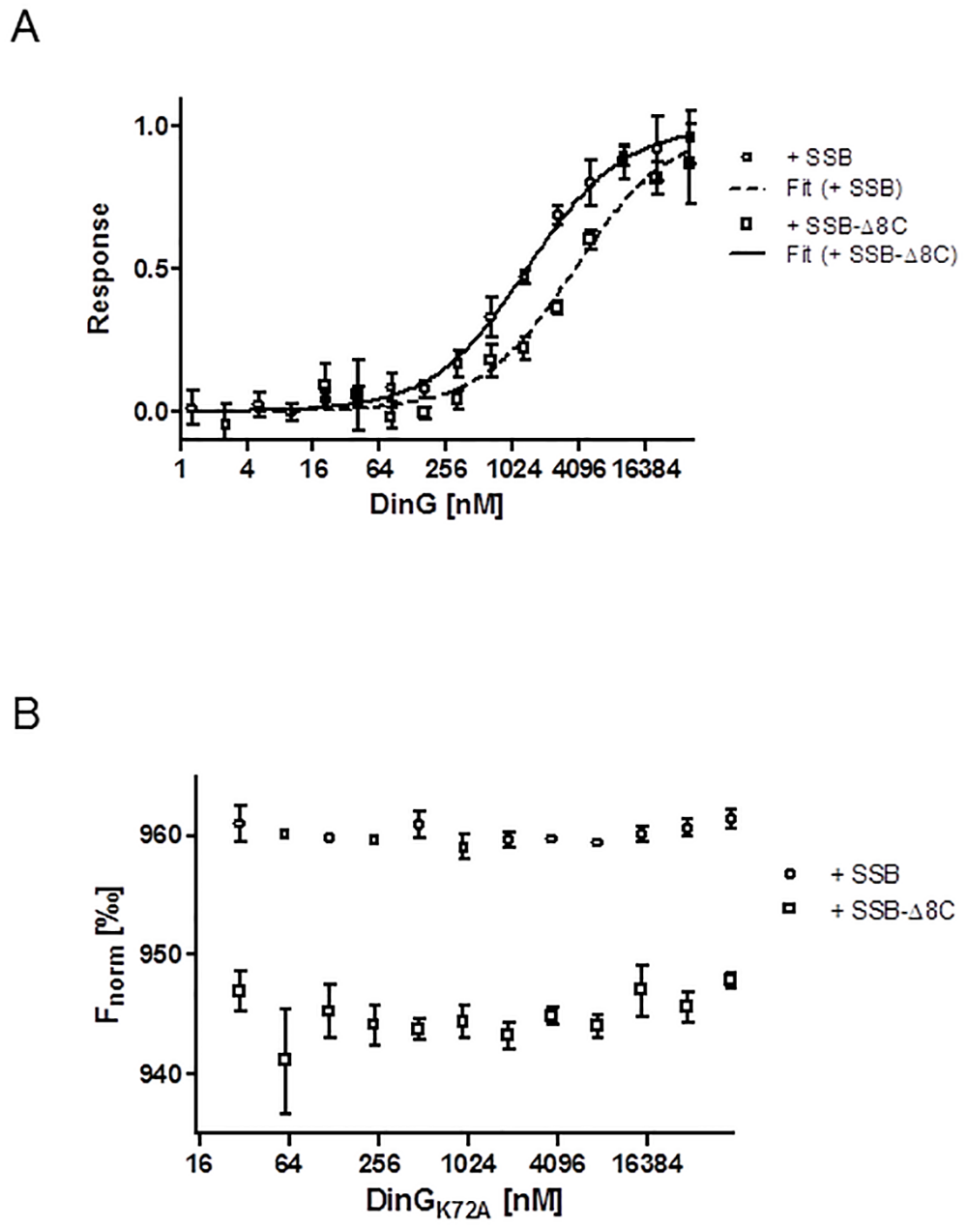
Protein interaction shown by microscale thermophoresis. A) MST of DinG_Nm_ with SSB_Nm_ and with SSB_Nm_Δ8C. The average and standard deviation of the normalised responses and the fitted curves are shown. B) MST of DinG_Nm_K72A with SSB_Nm_ and with SSB_Nm_Δ8C. No response could be detected and therefore the normalized fluorescence is plotted. The MST results for each protein combination include the data of three independent experiments.

The combination of DinG_Nm_ together with SSB_Nm_ resulted in the complete co-precipitation of DinG_Nm_ and SSB_Nm_. The MST assays with the SSB proteins as labelled molecules and the DinG proteins as ligands confirmed the interaction. The binding between SSB_Nm_ and DinG_Nm_ had a K_d_ value of 1.36 ±0.11 μM, and the binding between SSB_NmΔ8C_ and DinG_Nm_ had a K_d_ value of 3.71 ±1.03 μM (Fig 8A). The MST analyses of SSB_Nm_ or SSB_NmΔ8C_ with the mutant protein DinG_NmK72A_ did not yield any responses indicating no interaction (Fig 8B).

### NmΔ*dinG* cells are sensitive to DNA intrastrand crosslinking agents

**Fig 9:**
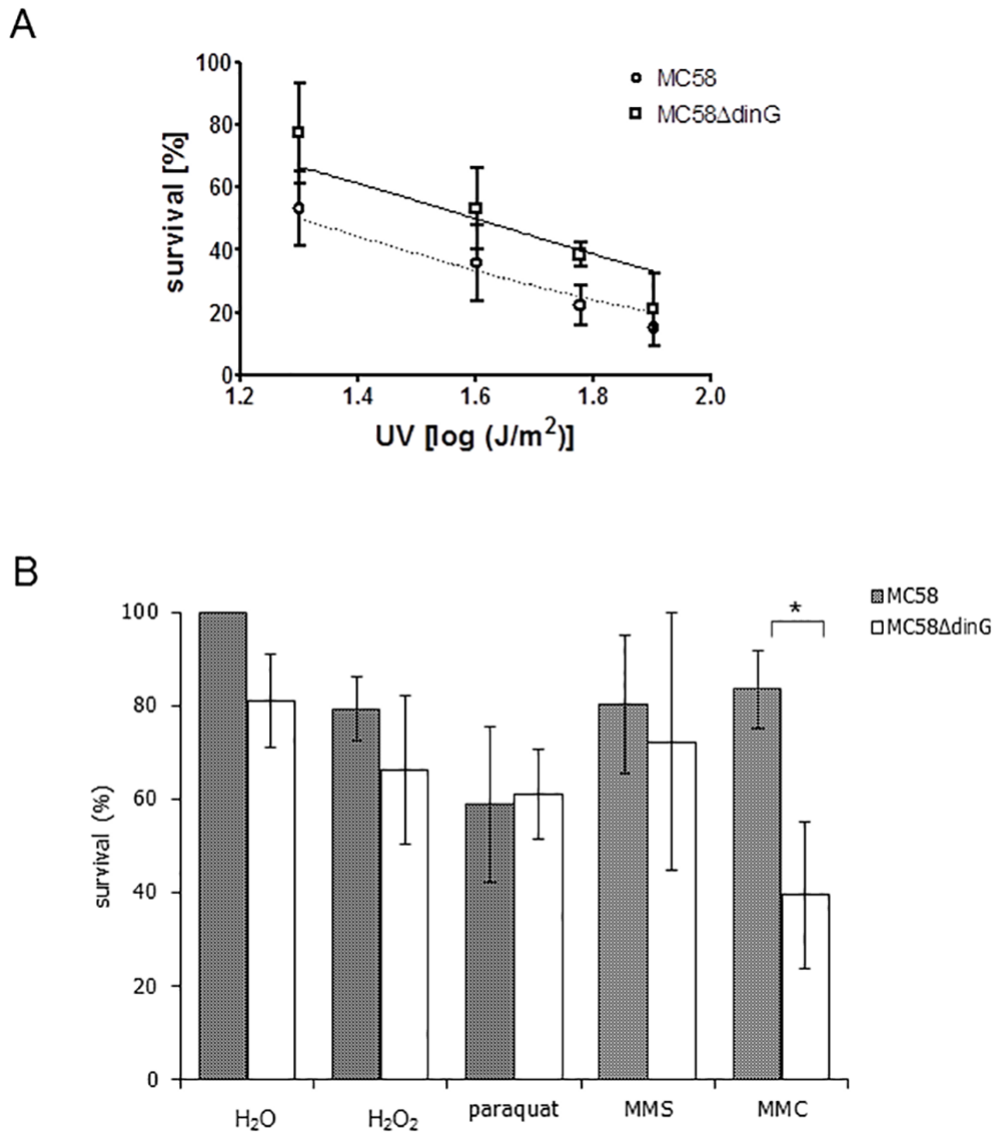
NmΔ*dinG* cells are sensitive to DNA intrastrand crosslinking agents. A) Survival rate of Nm MC58 wildtype (MC58 wt) and Δ*dinG*_*Nm*_ (MC58ΔdinG) after exposing the cells to the indicated UV fluences. B) Survival rate of Nm MC58 wt and MC58ΔdinG after treating the cells with 10 mM hydrogen peroxide (H_2_O_2_), 0.5 mM paraquat, 10 nM MMS or 10 ng/ml MMC as described in the Materials and Methods. The survival rate was calculated relative to the untreated wildtype. The results are from at least 3 independent experiments. A *p*-value < 0.01 is indicated by an asterisk.

The influence of genotoxic agents on Nm survival in the absence of the helicase DinG was examined by comparing Nm wildtype with NmΔ*dinG*. The Nm wildtype and NmΔ*dinG* were tested for sensitivity to UV irradiation, hydrogen peroxide (H_2_O_2_), paraquat, methyl methanosulphonate (MMS) and mitomycin C (MMC). When exposing the cells to increasing doses of UV the mutant cells survived slightly but not significantly better (IC_50_ = 40 J/m^2^) than the wildtype (IC_50_ = 20 J/m^2^) (Fig 9A). The NmΔ*dinG* mutant was equally sensitive to oxidative stress (H_2_O_2_ and paraquat) or alkylating stress (MMS) as the Nm wildtype (Fig 9B). The stress inflicted by MMC resulted in a significant difference in survival rates between wildtype and mutant, indicating a key role for DinG in repair of DNA double strand breaks caused by MMC (Fig 9B). This sensitivity of the mutant was confirmed in a similar experiment using bleomycin, another DNA double strand break causing agent (S6 Fig).

### Response of *N*. *meningitidis* wildtype and NmΔ*dinG* to DNA double strand break damage

**Fig 10:**
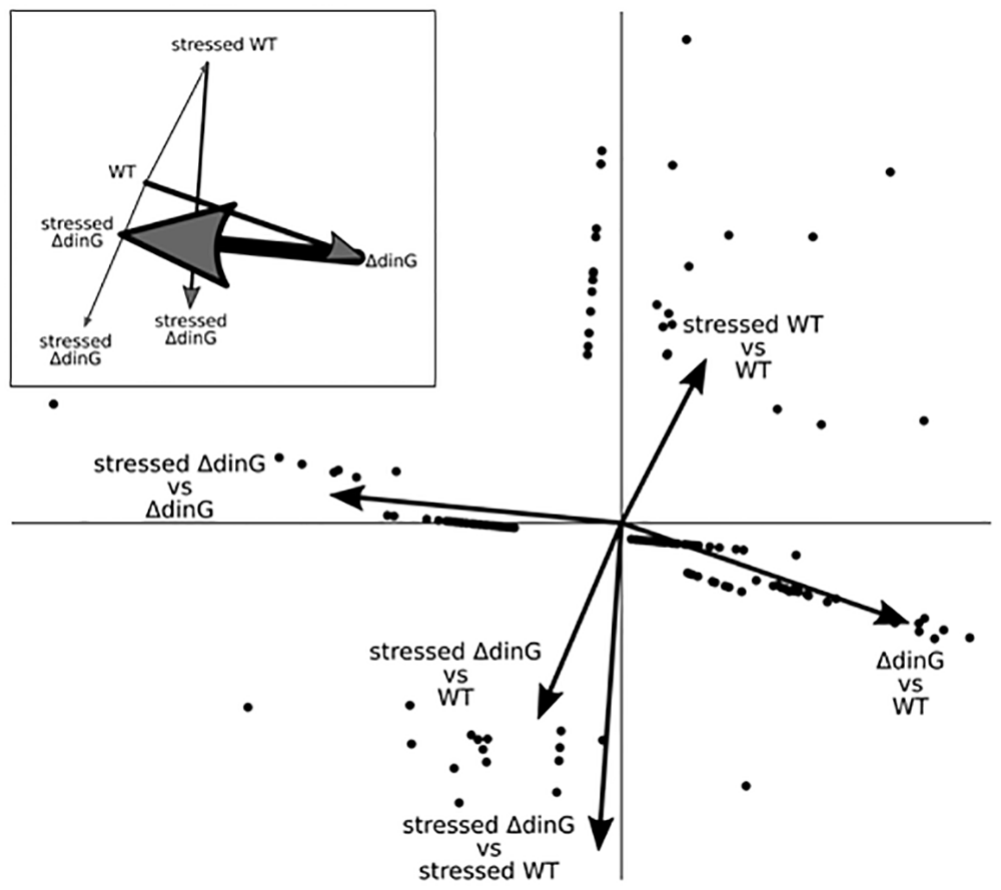
PCA of differential abundant proteins in Nm wildtype and NmΔ*dinG*. The PCA results of the DA proteins from S1 Table are shown in the form of a Variables Factor Map. Each point represents one gene listed in S1 Table, excluding the outlier Opc. The insert shows the vectors weighted by number of DA proteins and with their tails moved to the corresponding head.

Since the MMC induced genotoxic stress led to a significantly lower survival rate of NmΔ*dinG*, we further investigated the protein expression profiles of the Nm wildtype and mutant constitutively and under MMC stress. Quantitative mass spectrometry-based protein profiling was employed to identify differentially abundant (DA) proteins. A good overlap of detected proteins was reached (S2 Fig). When disrupting *dinG* in Nm (NmΔ*dinG*), 48 DA proteins were identified with 9 proteins more and 39 proteins less abundant when compared to the wildtype (S1 Table). Under normative condition, in the NmΔ*dinG* the proteins MinC and NuoE were found to be less abundance than in wildtype, by 3.4 and 2.1 fold, respectively. Parallel to this a more than 3 fold increase was seen in the amount of the SOS response repressor LexA homologue NMB0556. Under stress condition this protein was less abundant in the NmΔ*dinG* cells by 1.6 fold (S1 Table). The highest number of DA proteins was observed when comparing the MMC stressed NmΔ*dinG* to the unstressed NmΔ*dinG* cells. We found 82 more abundant and 52 less abundant proteins in MMC stressed NmΔ*dinG* compared to the unstressed NmΔ*dinG*. The proteins MinC and NuoE were found to be more abundant in stressed NmΔ*dinG* by 4.2 and 2.2 fold, respectively (S7 Table). Interestingly, 70% (27out of the 39) of the more abundance proteins in unstressed NmΔ*dinG* (column H in S1 Table) were found to be less abundant in MMC stressed NmΔ*dinG* (column O in S1 Table). In addition, 89% (8 out of 9) of the less abundant proteins were more abundant (columns G and P in S1 Table). This shows the general opposite tendencies of the effects of *dinG* deletion and MMC stress on the mutant. These opposing tendencies were confirmed by Principle Component Analysis (PCA) (Fig 10). The PCA was done using R with the package FactoMineR [19, 20].

**Fig 11:**
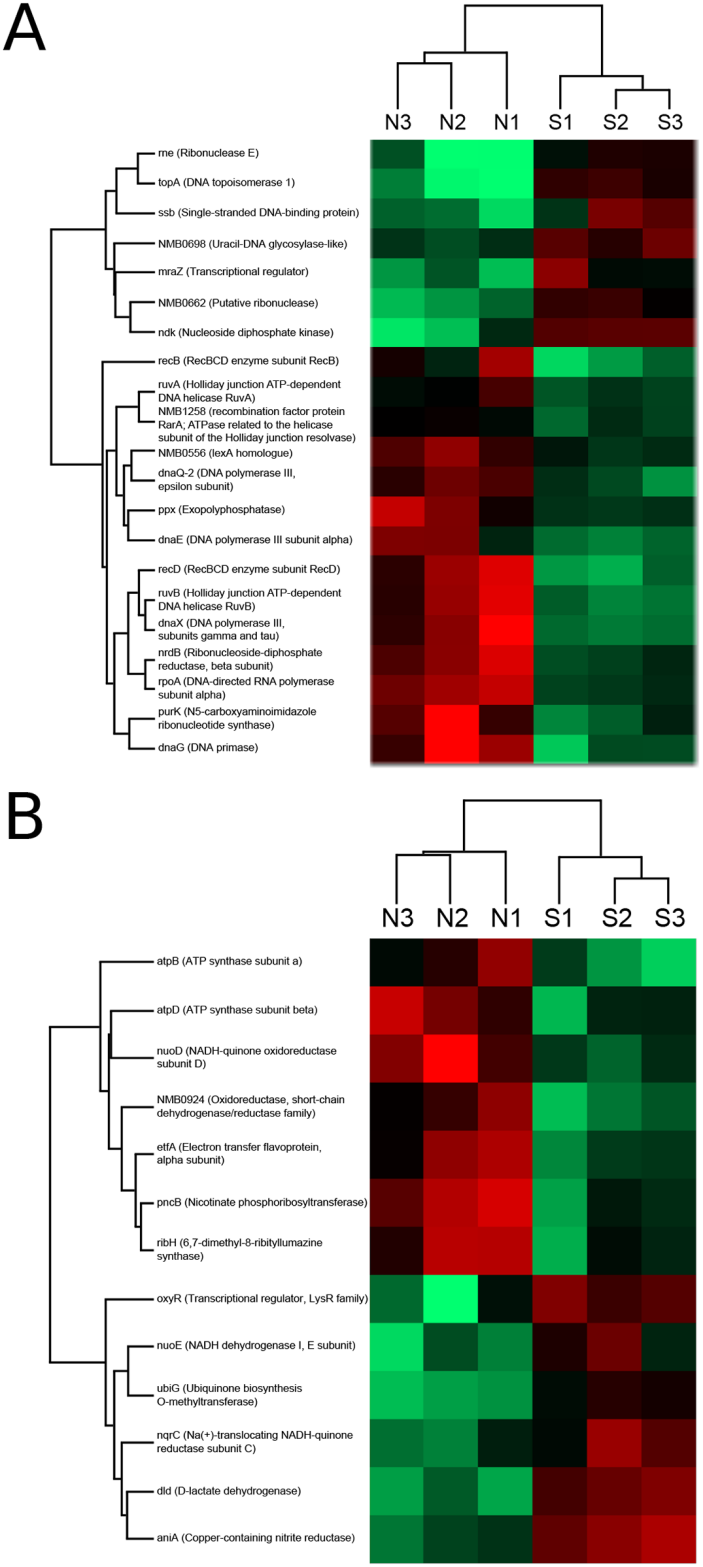
Heatmaps for differential abundant proteins in NmΔ*dinG*. The abundances of proteins belonging to two different groups, A) DNA metabolism, and B) electron transfer, are shown. Gene and protein names are given on the left and the three experiments for the native condition (N) and the MMC stress condition (S) are indicated on top.

In addition to the amino acid and carbon metabolism, including 2-oxocarboxylic acid metabolism, the proteins involved in DNA metabolism are one of the most affected categories in the stressed NmΔ*dinG*. The changes for the DA proteins involved in DNA metabolism are shown in Fig 11A. The list of these proteins includes eight less abundant proteins, namely DnaG, DnaQ-2, DnaX, DnaE, RecB, RecD, RuvA and RuvB, and the two more abundant proteins TopA and SSB (S1 Table). The DA proteins involved in electron transfer and therefore in the maintenance of the redox state of the cell are shown in Fig 11B, with AniA being a central electron acceptor up-regulated under MMC stress condition.

### DinG_Nm_ does not affect transformation or replication in *N*. *meningitidis*

Nm is naturally competent for transformation throughout its lifecycle, and this feature plays a major role in genome maintenance in *Neisseria spp*. Therefore, we quantified the transformation efficiency in NmΔ*dinG*. No significant difference between wildtype and NmΔ*dinG* in transformation was detected (S3 Fig). As helicases might play a role in replication of DNA, the DNA content and the protein mass of neisserial cells were measured by flow cytometry (S4 Fig). Due to the pathogenic potential of Nm, for this experiment *Neisseria gonorrhoeae* (Ng) strain MS11 and the NgΔ*dinG* mutant were used to determine the chromosome equivalents and the number of active replication forks per cell. For exponentially grown cultures, no difference in the number of active replication forks was detected, and the total DNA contents of the wildtype and mutant cells were similar (S4 and S5 Figs). Upon treatment with rifampicin (RIF) and cephalexin (CPX), the DNA content of the Ng MS11 wildtype and NgΔ*dinG* cells did not differ significantly (S4C and S4D Fig).

## Discussion

The bacterial helicase DinG is homologous to the archaeal and eukaryotic helicases XPD/Rad3 and FANCJ. They all belong to the SF2 helicase with an intrinsic 5′ → 3′ helicase activity and a characteristic Fe-S binding domain [6]. The role of eukaryotic XPD in nucleotide excision repair is well recognized [21, 22], but the role of the bacterial DinG and archaeal XPD in DNA repair is not well understood. In this study, we characterized the neisserial helicase DinG to understand its role in DNA repair, transformation and replication.

The Expression of *dinG*_Ec_ was found to be induced by MMC, which induces DSBs due to interstrand cross-links in DNA [23]. The stress experiments presented here showed that loss of *dinG* in Nm severely reduces the survival of the cells when exposed to MMC. On the other hand, the survival of NmΔ*dinG* cells was not significantly reduced due to stress inflicted by UV, H_2_O_2_, paraquat or MMS. We suggest that DinG_Nm_ plays a role in maintaining genome integrity, especially when encountering severe forms of genotoxic stress, such as DSBs.

Our comparative quantitative proteomics revealed differences in the expression level of proteins in NmΔ*dinG* compared to the Nm wildtype. Eighteen of the thirty nine proteins more abundant upon *dinG* deletion are involved in central housekeeping functions, such as amino acid, carbon and nucleic acid metabolisms. This suggests that there might be a general change in the regulation of the metabolism in response to deletion of *dinG*.

Opc, a phase variable outer membrane protein and an effective invasin for human endothelial cells, was down regulated when comparing the mutant with the wildtype. This is pointing to a random phase switch in the gene which happened during the cloning of the mutant and can also be attributed to the selection of an opacity negative colony during cloning. The generally lower expression of Opc in the mutant is therefore a none-stress related result [24–26].

MinC was found to be less abundant in the mutant compared to the wildtype by 3.4 fold. MinC is a cell-division inhibitor preventing the formation of the Z ring and is essential for proper cell division in cocci [27, 28]. Two proteins involved in the electron transport chain (NuoE, CycP) were found to be more abundant in mutant than the wildtype. Only two DNA metabolic proteins, namely DnaG and RecD, were found to be more abundant in the mutant. The DnaG, a DNA primase, is involved in short strand RNA synthesis during DNA replication. RecD is a helicase in the enzyme complex RecBCD, which is involved in conjugal and transductional recombination, DNA repair, and degradation of foreign DNA [29]. Thereby, in the absence of DinG, RecD is the only DA protein found having a comparable and possibly compensatory activity.

Co-transcription of genes in an operon could lead to a symmetric differential expression of proteins. For three out of seven genes in operons (S1 Table) this symmetry was found [30]. Interestingly, this was not the case for *minC* and *oxyR* although OxyR negatively regulates the promoter in the *minCDE-oxyR* gene cluster [31]. OxyR, a regulator for the expression of catalase [32], is overexpressed in NmΔ*dinG* under the MMC stress condition. Although an association of MMC with oxidative stress (OS) was shown in eukaryotic systems [33], a homologue to xanthine dehydrogenase, an enzyme responsible for oxygen radical generation [34, 35], was not identified in *Neisseria sp*. This still leaves the possibility of another enzyme exerting this activity, possibly an oxidoreductase involved in the electron transport chain [36]. With the OS signal provoked by MMC the increase in OxyR would lead to an activation of the catalase gene (*kat*) and thereby an alleviation from the OS.

Cytochrome c′ was less abundant in the NmΔ*dinG* mutant while AniA, a nitrite reductase, was more abundant under MMC stress. Both proteins participate in the electron transport chain and are involved in the defence against nitrosative stress [37, 38].

Three of the DA proteins (BfrA, BfrB, GshB) take part in the defence against oxidative stress and OxyR is a regulator of the oxidative stress response [39]. In addition many proteins involved in the electron transport, which ultimately feeds into the production of ROS, are differentially abundant (S1 Table). While iron is an important co-factor for proteins involved in the electron transport, only a few (4) proteins directly regulated by Fur were DA with NMB1395, an alcohol dehydrogenase, being 3.8 times more abundant in stressed NmΔ*dinG* compared to unstressed NmΔ*dinG* (S1 Table) [40]. Although no unilateral direction of up- or down-regulation is visible, all these changes may reflect the general adaption of the system to cope with ROS and also to reduce the overall oxidation state of the cell. The general change in regulation of genes when the Nm bacterium is under stress, like in the blood, comprise that the genes involved in energy metabolism are up-regulated and house-keeping genes are down-regulated [41].

Bacteria respond to DNA damage by mounting a coordinated cellular response, governed by the RecA and LexA proteins, called SOS response, by down-regulating more than 40 unlinked genes [42]. The inactivation of LexA by activated RecA-ssDNA filaments causes the expression of genes that increases DNA damage repair and tolerance [43]. The Nm-genome lacks the conserved LexA binding site (SOS box) [44]. However, recently a LexA orthologue (GenBank ID: **NGO1427**) has been identified in Ng and is found to be modulated by oxidation [45]. Here, the Nm orthologue (GenBank ID: **NMB0556**) was found to be more abundant (by 3 fold) in the unstressed NmΔ*dinG*, but under MMC stress it was less abundant (by 1.6 fold). At present, it is not known how this orthologue regulates the SOS response under MMC stress in the absence of the canonical SOS box, but our results point to a significant impact.

Looking at the DA proteins detected when comparing stressed and unstressed NmΔ*dinG* we found that two proteins, TopA and SSB, were more abundant in the stressed NmΔ*dinG* (S1 Table). The function of topoisomerase I is required for efficient transcriptional activation of the *recA* and *dinD1* promoters during the *E*. *coli* SOS response to trimethoprim or MMC [46]. Recently, it was also shown that topoisomerase I interacts with RecA with ATP as an enhancing factor [47]. SSB is known to bind ssDNA and interacts with more than 15 other proteins [48]. An *in vitro* study revealed that *E*. *coli* SSB was able to form a stable protein complex with DinG and stimulate its DNA helicase activity [49]. On the other hand a study in *B*. *subtilis* failed to see interactions between DinG and SSB in pulldown experiments [50]. Our analyses revealed that DinG_Nm_ interacts with SSB_Nm_ with a value that can be considered to be in the range between stable and transient interactions [51].

Eight DNA metabolic proteins were less abundant in MMC stressed NmΔ*dinG* cells when compared to the unstressed mutant (S1 Table). Among them, the gene products of *dnaE*, *dnaX* and *dnaQ-2* are subunits of the DNA polymerase III holoenzyme. An interaction network analysis in *E*. *coli* revealed that Pol III subunits and clamp loader co-purify together with TopA and PriA helicase [52]. Several studies have reported the direct binding of the *E*. *coli* SSB to the Pol III holoenzyme [53–55]. This shows an interaction network of proteins involved in diverse biological process and has been proposed to be important for stabilization of the replication machinery and for facilitating Okazaki fragment replication initiation [53–55]. Other less abundant DNA metabolic proteins in MMC stressed NmΔ*dinG* cells were RuvA, RuvB, RecB, RecD and DnaG. RuvA and RuvB together with RuvC form a complex and play important roles in the homologous genetic recombination and the recombinational repair of damaged DNA [56]. In *E*. *coli*, the *ruvA* and *ruvB* genes are SOS inducible and regulated by LexA repressor [57]. DnaG and RecD are two DNA metabolic proteins that are inversely regulated in stressed and unstressed NmΔ*dinG*. These observations reveal that, especially under stress, DinG is involved in the recombinational repair pathway as the lack of it leads to a deregulation of compounds of the DSB repair system.

Only a few studies tried to pinpoint the role of DinG helicase in DNA repair pathways, and suggested that its possible involvement in replication and recombinational repair [8, 10, 58]. The study by Boubakri *et al*. suggested that DinG_Ec_ enables the replication fork to move along transcribed DNA by unwinding R-loops or displacing the transcribing RNA polymerase in conjunction with UvrD or Rep helicase [8]. DinG_Nm_ might play a similar role or might help remove other structures that inhibit progression of DNA replication.

The DinG helicase and its homologues contain four conserved cysteine residues. These residues are ligands for a Fe-S cluster, which is redox active and DinG_Ec_ is fully active only under oxidizing conditions [59]. We show here that DinG_Nm_ also contains four iron molecules per protein molecule for a [4Fe-4S] cluster. It was recently suggested that helicases with an Fe-S binding domain might unwind non-conventional DNA or RNA structures or displace proteins from cognate DNA binding sites, thus relieving stalled DNA replication or transcription complexes [60]. This ability would make DinG_Nm_ into an oxidation state dependent transcriptional regulator.

In summary, our data indicate an important role for DinG_Nm_ in DSB repair. Nm cells lacking DinG helicase show, compared to the wildtype cells, the de-regulation of many proteins. Under MMC stress this de-regulation is inversed and over compensated. The regulation includes mainly proteins which are involved in energy, amino acid and nucleotide metabolism, and DNA replication and repair. Future studies on the functions and interactions of DinG_Nm_, also focusing on the redox state of the cells under stress, will reveal more insights into its cellular roles and thus show the part this helicase takes in DNA repair pathways preserving the integrity of the genome.

## Materials and methods

### Bioinformatics analysis

Sequence data for *dinG* was obtained from the PubMLST database [61] and from NCBI [17]. The *dinG*_*Nm*_ nucleotide sequences were searched for occurrences of the DNA uptake sequence (DUS) and single nucleotide polymorphisms, and in the deduced DinG_Nm_ amino acid sequence structural helicase motifs were identified by alignment with the sequence for DinG_Ec_. Sequence conservation was calculated using plotcon from the EMBOSS package [62] and visualized on the protein structure using ConSurf [63]. The multiple sequence alignment grid profile was extracted using UGENE [64] and the prediction of functional effects of sequence variants was done with SNAP2 [65].

### Bacterial strains and growth conditions

*Neisseria* strains were grown on GC agar plates or in liquid GC medium supplemented with IsoVitaleX at 37°C and 5% CO_2_. When required, kanamycin at a final concentration of 100 μg/ml was added. *E*. *coli* strains were grown at 37°C on LB plates or in LB medium containing kanamycin (50 μg/ml). The bacterial strains and plasmids employed in this study are listed in Table 1.

**Table 1:** Bacterial strains and vectors.

**strain**	**remarks**	**reference**
MC58	*N*. *meningitidis* wildtype, serogroup B	[66]
McSAF93B	MC58 derivative, Δ*dinG*, kanamycin resistant	this study
M1080	*N*. *meningitidis* wildtype, serogroup B	[67]
M400 (M1080-A)	M1080 derivative, inducible *recA* (*recA6*)	[68, 69]
MS11	*N*. *gonorrhoeae* wildtype	[70]
N400	MS11 derivative, inducible *recA* (*recA6*)	[71]
ER2566	*E*. *coli*	NEB
NiCo21 (DE3)	*E*. *coli*	NEB
**vector**	**remarks**	
pJet1.2	cloning vector, *bla*	Fermentas
pET-28b(+)	expression vector	Novagen
pQE-30	expression vector	Qiagen
pUP6	source for *aph* from Tn5, derivate of pHSS6 (ACCESSION: M84115), carries two DNA uptake sequences	[72]
pSAF93B	vector to generate the *dinG* K.O. mutant	this study
pOHA-D1	vector used in the transformation assay	[16]
pSB13	DinG_Nm_ expression vector	this study
pSAF92	DinG_NmK72A_ expression vector	this study
pEH1	Ssb_Nm_ expression vector	[73]
pSAF104	Ssb_NmΔ8C_ expression vector	this study

### Cloning of *Neisseria meningitidis* SSB, SSB_NmΔ8C_, *dinG*_Nm_ and *dinG*_NmK72A_

The genes were PCR amplified from genomic DNA isolated from *N*. *meningitidis* MC58 using the appropriate primers (S2 Table). The PCR product was digested with the restriction enzymes *Xba*I and *Sal*I and ligated into a identically digested pET-28b(+) vector (Novagen). The resulting plasmid pSB13 coding for a protein with a C-terminal 6xHis-tag was transformed into *E*. *coli* NiCo21 (DE3) (NEB) containing the plasmid pLysS (Novagen). The point mutation in the ATP binding motif (K72A) was created in pSB13 using site-directed mutagenesis with the primers SF177 and SF178 resulting into pSAF92. Cloning of SSB_Nm_ has been described earlier [74]. Primers SF275 and SF276 were used to amplify the vector pSAF104 using the vector pEH1 as a template to obtain the *ssb*_NmΔ8C_ construct.

The sequences of all constructs were verified using appropriate sequencing primers.

### Over-expression and purification of recombinant proteins

*E*. *coli* NiCo21(DE3) cells carrying the expression plasmid (Table 1) were grown in LB medium containing 0.5 M sorbitol, 2.5 mM betaine, 1% glucose, 50 μg/ml kanamycin and 20μg/ml chloramphenicol at 37°C until OD_600nm_ ≈ 0.4, then the temperature was reduced to 18°C. 1 mM isopropyl *β*-D-thiogalactopyranoside (IPTG) was added and the culture incubated overnight. Cells were harvested, resuspended in lysis buffer (10 mM immidazole, 50 mM NaH_2_PO_4,_ 300 mM NaCl, 20 mM β-mercaptoethanol, Complete Protease Inhibitor (Roche), 2 mM MgCl_2_, Benzonase (Novagen)) and disrupted by sonication. Cleared lysate was loaded onto a Ni-NTA column (Qiagen) and the column was washed according to the manufacturer’s instructions. Bound proteins were eluted with buffer containing 250 mM imidazole. The fractions containing the recombinant protein were dialysed overnight against dialysis buffer (20 mM Tris-HCl pH 7.5, 100 mM NaCl, 2 mM dithiothreitol (DTT), and subjected to further purification by ion exchange chromatography using a Resource Q column (GE Healthcare) equilibrated with a buffer containing 20 mM Tris pH 7.5, 100 mM NaCl, 2 mM DTT. Proteins were eluted with an increasing ionic strength gradient up to 1 M NaCl. Fractions with pure protein were dialyzed against 20 mM Tris pH 7.5, 600 mM NaCl, 2 mM DTT, pooled and concentrated by ultrafiltration (Amicon). The protein was stored in 20 mM Tris-HCl pH 7.5, 300 mM NaCl, 10% glycerol at -80°C. The over-expression and purification of SSB_Nm_ and SSB_NmΔ8C_ were done as described earlier [74].

### ATPase, DNA binding and unwinding assays

All DNA oligonucleotides used in this study were purchased from Operon Biotechnologies, Inc., and the sequences were adopted from previous studies [75]. DNA substrates were prepared as described elsewhere [76]. The oligonucleotides used in this study are given in S2 Table. The DNA binding, unwinding and ATPase assays were carried out as described previously [75] with slight modifications. **i) ATPase assays**: DinG_Nm_ or DinG_NmK72A_ was added to initiate a 10 μl reaction in the presence of 100 nM DNA cofactor in ATPase buffer [20 mM Tris/HCl (pH 7.5), 2 mM MgCl_2_, 100 μg BSA/ml, 25 mM cold ATP, 0.023 nM [γ-^32^P]ATP, 2 mM DTT]. Also reactions containing DNA cofactor but without DinG_Nm_ protein, and DinG_Nm_ but without DNA cofactor were included per experiment. The reaction mixture was incubated at 37°C for 30 min and terminated by adding 5 μl of 0.5 M EDTA (pH 8.0). Samples (2 μl) were spotted onto TLC plates (PEI Cellulose F, Merck) at 1.5 cm intervals and resolved using a solution containing 1 M formic acid and 0.5 M LiCl. **ii) DNA binding assays**: Reaction mixtures (20 μl) contained 0.1 nM γ-^32^P-labelled DNA substrates, binding buffer [40 mM Tris-HCl, (pH 8), 2.5 mM EDTA, 2 mM MgCl_2_, 100 mg/ml bovine serum albumin (BSA), 6% glycerol, and 2 mM DTT] and the indicated concentrations of the DinG_Nm_ or DinG_NmK72A_ protein. After incubation for 15 min on ice, 2 μl of 60% glycerol was added to the reaction immediately before loading on to a 30 min pre-run 5% native PAGE gel (29:1, acrylamide: bisacrylamide). Electrophoresis was done using low ionic strength buffer (6.7 mM Tris HCl pH 8, 3.3 mM sodium-acetate pH 5.5 and 2 mM EDTA pH 8) at 200 V for 5 min followed by 160 V for 85 min in ice water bath with continuous buffer recirculation between the upper and lower chambers. **iii) Unwinding assays**. All helicase unwinding reactions (10 μl) were carried out in helicase reaction buffer (20 mM Tris-HCl (pH 7.5), 50 mM NaCl, 2 mM DTT, 2 mM MgCl_2_, 2 mM ATP and 50 mg/ml BSA). 0.1 nM γ-^32^P-labelled DNA substrate was mixed with increasing concentration of DinG_Nm_ or DinG_NmK72A_ and incubated at 37°C for 30 min. The reaction was terminated by adding 5 μl of 3× stop dye (50 mM EDTA, 40% glycerol, 0.9% SDS, 0.1% bromophenol blue and 0.1% xylene cyanol) along with 10× molar excess unlabelled oligonucleotide complementary to the unlabelled strand in the substrate. The reaction products were analysed on 8% native polyacrylamide (19:1) gel containing 0.1% SDS in 1× Tris/borate/EDTA buffer.

### Ammonium sulfate co-precipitation

Co-precipitation experiments were performed as described before [77]. Briefly, 20 μM of DinG_Nm_ was incubated with 80 μM SSB_Nm_ in 20 μl co-precipitation buffer (10 mM Tris-HCl, pH 7.2, 300 mM NaCl, 10% [v/v] glycerol) on ice for 15 min. 10 μl of ammonium sulphate solution (450 g/l) was added, the reaction incubated on ice for 15 min, and then centrifuged for 1 min at 18,000×g. The pellets were washed three times with 50 μl co-precipitation buffer including 150 g/l ammonium sulphate and then dissolved in 50 μl 1× NuPAGE LDS sample buffer (Thermo Fisher Scientific). 10% polyacrylamide gels were loaded with 10 μl sample/well, run at 15 V/cm and stained with Coomassie brilliant blue.

### Microscale thermophoresis

Microscale thermophoresis (MST), a method for measuring molecule interaction, is described extensively elsewhere [78]. Labelling of SSB_Nm_ was carried out following the manufacturers’ instructions using the Monolith NT Protein Labeling Kit RED–NHS (NanoTemper Technologies GmbH) resulting in a degree of labelling (DOL) of 0.7. Different concentrations of DinG_Nm_ where incubated with 20.7 nM SSB_Nm_ in 20 mM HEPES buffer (pH 7.5) containing 300 mM NaCl, 0.05% Tween 20, 0.1% Pluronic F-127, 0.1% PEG 8000 and 2 mM DTT. Samples were immediately loaded into Premium coated capillaries (NanoTemper Technologies GmbH) and measured at 22°C and 20% MST power in a Monolith NT.115 series instrument (NanoTemper Technologies GmbH).

### Iron chelation assay

The amount of iron present in purified protein was determined by using bathophenanthroline as described earlier [17]. Briefly, 50 μl of a known amount of protein was denatured using 15 μl of 38% HCl at 100°C for 15 min, and centrifuged to pellet insoluble material. The control reaction contained only protein storage buffer. The supernatant was collected, neutralized with 650 μl, 0.5 M Tris-HCl (pH 8.5) and freshly prepared 50 μl of 5% ascorbic acid and 200 μl of 0.1% [w/v] bathophenanthroline disulfonic acid disodium salt (Sigma) were added. After incubation at room temperature for 1 h, absorbance was measured at 535 nm. The iron concentration was calculated by employing a standard curve set up with ferrous ammonium sulfate hexa hydrate of known concentration (Sigma). The assay was performed three times.

### Construction of the NmΔdinG mutant

To generate the NmΔ*dinG* mutant, a vector was constructed to be used in transformation for replacing *dinG* with the marker gene *aph*. The vector backbone was amplified by PCR with the primers JEE61 and JEE62 with vector pJet1.2 as template. The *aph* was amplified with the primer 8184OHA_AphEcoRI_REV and 8186DUS_AphNheI_FOR from pUP6. Sequences adjacent to *dinG* were amplified with the primer pairs SF179/SF180 and SF181/SF182 from genomic DNA from MC58 (Table 1). Gibson assembly was performed to combine the sequences and the product was transformed into *E*. *coli* ER2566. Positive clones were selected for by growth on agar plates containing 50 μg/ml kanamycin and 100 μg/ml ampicillin. The correctness of the final plasmid pSAF93B was confirmed by restriction digest and sequencing (Table 1). The plasmid pSAF93B was transformed into the Neisseria strains MC58, M1080, M400, MS11 and N400 by natural transformation using kanamycin resistance for the selection. Positive clones were checked by PCR for proper insertion of the *aph*.

### DNA damage sensitivity assays

Nm cells from overnight plate culture were suspended in liquid GC medium to OD_660_ ≈ 0.3, and diluted 10 times in CO_2_ saturated GC medium containing IsoVitaleX. The cells were allowed to grow for two hours at 37°C with tumbling. Then the cells were treated separately with the following chemicals; 100 μM H_2_O_2_ [3], 0.5 mM paraquat [3], 10 mM MMS [3] or 10 μg/l MMC [79]. After additional growth for 1 h with tumbling at 37°C, a tenfold serial dilutions were prepared in PBS and 50 μl aliquots of the 10^−5^ and 10^−6^ dilutions were spread out on GC agar plates. To test sensitivity to ultraviolet radiation, 50 μl aliquots of the 10^−5^ and 10^−6^ dilutions of untreated cells were spread on GC agar plates, irradiated at UV intensities of 0–80 J/m^2^ by using a CL-1000 Ultraviolet cross linker (Upland America). Finally, the plates were incubated overnight at 37°C with 5% CO_2_ for 12 to 18 h. Colonies were counted and survival rates were calculated as the ratio of the number of colony forming units (CFU) from treated to non-treated cells. The MMC treated samples were also subjected to quantitative mass spectrometry analyses.

### Proteomics analyses

Peptide characterization and quantitation were performed by electrospray-based high resolution mass spectrometry using a Q-Exactive instrument (Thermo Scientific). A detail description of sample pre-treatment, preparation and mass spectrometry analysis can be found in the S1 Text.

### Quantitative transformation assay

Quantitative transformation was performed essentially as previously described [12,52]. Briefly, *N*. *meningitidis* cells were grown on GC plates overnight at 37°C and suspended in CO_2_ saturated GC medium containing IsoVitaleX and 7 mM MgCl_2_. A 500 μl of cell suspension was mixed with 5 μl of plasmid DNA (pOHA-D1, 100 ng/μl) and incubated at 37°C for 15 min without agitation. In order to degrade extracellular DNA, benzonase (25 U/ml) was added and incubated for 10 min. Then 4.5 mL of pre-warmed CO_2_ saturated GC medium was added and the culture incubated at 37°C with tumbling for 4.5 h. Of each sample, 50 μl aliquots were spread on GC agar plates containing 8 μg/ml erythromycin and 100 μl of the 10^−5^ and 10^−6^ samples diluted in PBS were spread on plain GC agar plates. Following overnight incubation at 37°C and 5% CO_2_ colonies were counted. Transformation frequencies were calculated as the number of antibiotic-resistant colony forming units (CFU) per total CFU. The experiments were repeated at least three times.

## Supporting information

S1 Fig Helicase motifs of DinG_Nm_. (TIF)

S2 Fig Overlap of proteins identified by mass spectrometry analysis. (TIF)

S3 Fig DNA transformation is independent on the DinG helicase. (TIF)

S4 Fig Flow cytometry analysis of *N*. *gonorrhoeae* wildtype and NgΔ*dinG* cells. (TIF)

S5 Fig Loss of DinG helicase does not influence replication efficiency. (TIF)

S6 Fig Survival of cells under genotoxic stress causing double strand breaks. (TIF)

S1 Table List of differentially abundant (DA) proteins. DA proteins with significant differences in the comparisons as shown on top are listed by their gene names and gene NMB number. Values for down-regulation and up-regulation are shown in separate columns with the count on top. Colour coding for the values from high (red) to low (green) was used for the data cells.
(XLSX)

S2 Table Oligonucleotides. (PDF)

S1 Text Materials and methods. (PDF)
