# Supplementary figures and images for: The helicase DinG responds to stress due to DNA double strand breaks
Source: PLoS One. 2017 Nov 9;12(11):e0187900. doi: 10.1371/journal.pone.0187900 (PMC5679670; doi:10.1371/journal.pone.0187900)

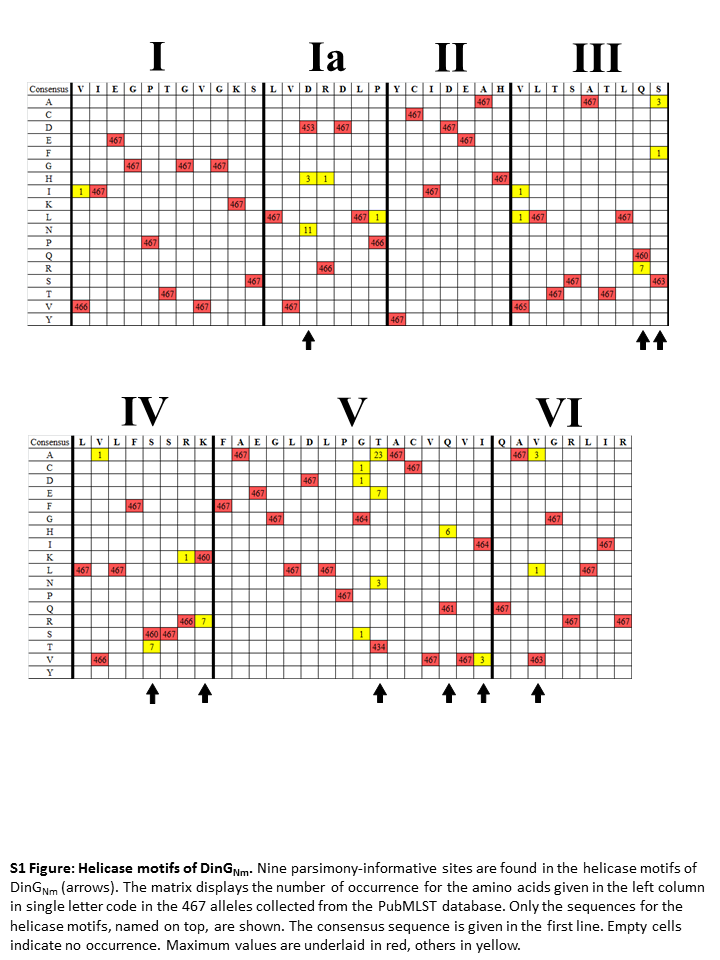

Supplement: S1 Fig — (TIF) [file pone.0187900.s001.TIF]

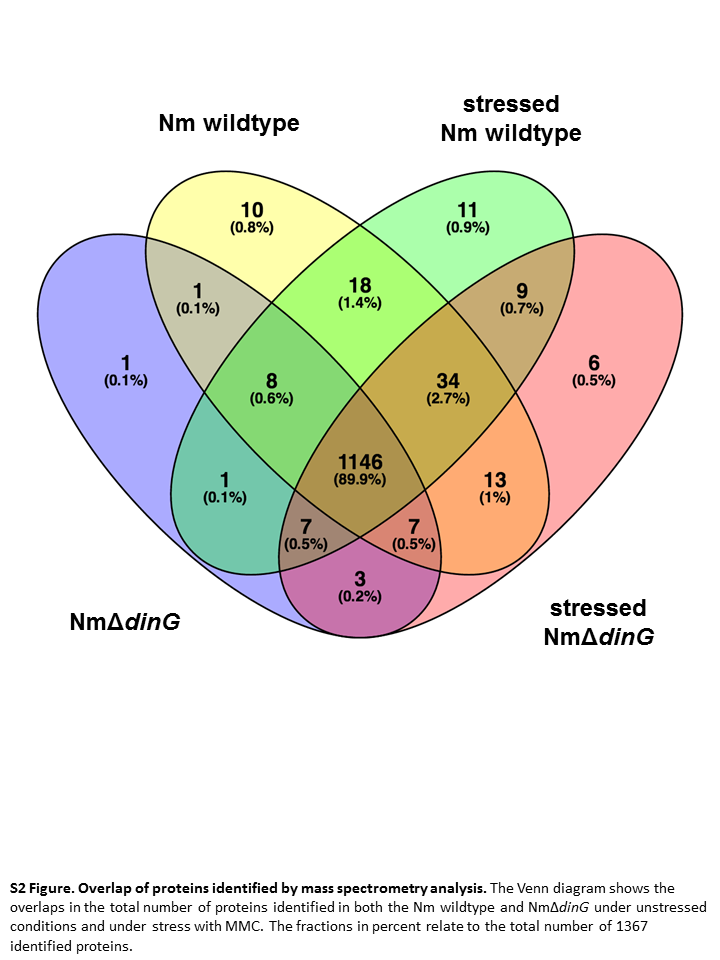

Supplement: S2 Fig — (TIF) [file pone.0187900.s002.TIF]

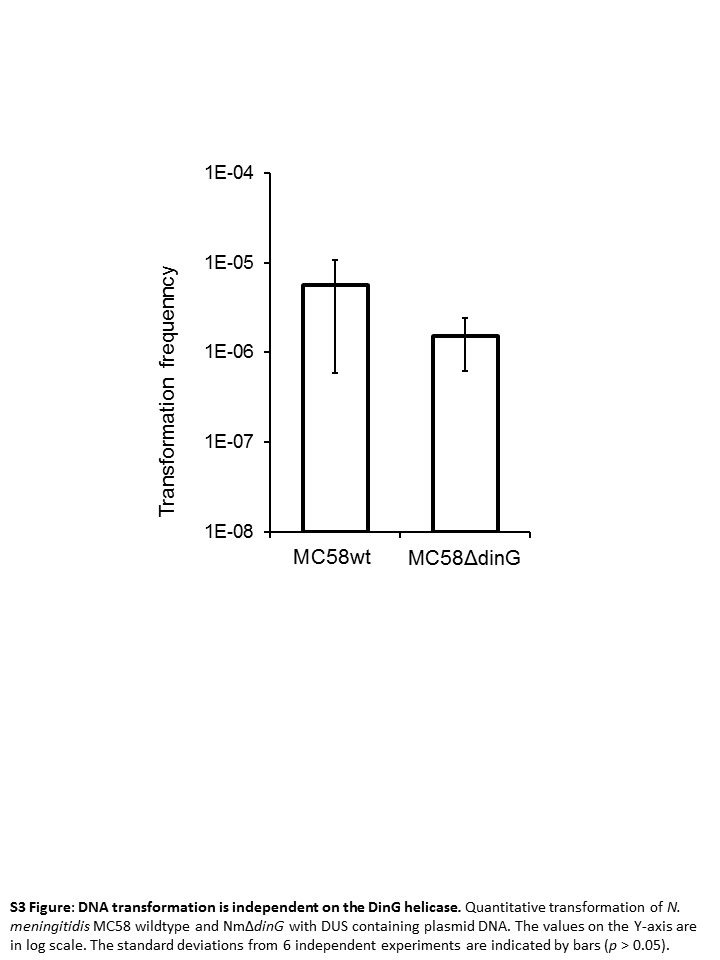

Supplement: S3 Fig — (TIF) [file pone.0187900.s003.TIF]

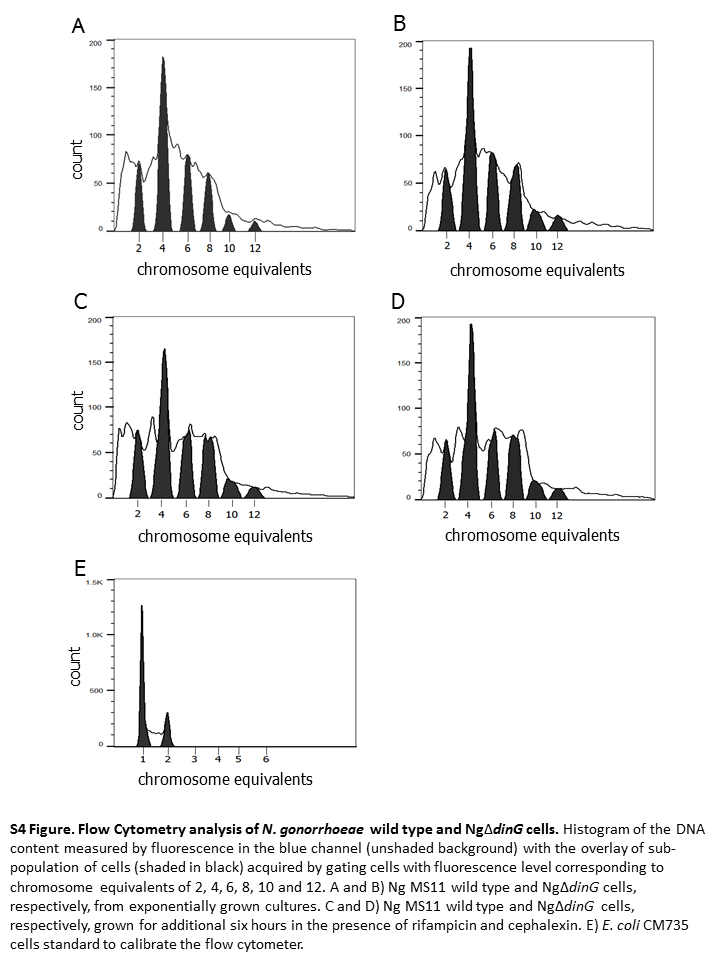

Supplement: S4 Fig — (TIF) [file pone.0187900.s004.TIF]

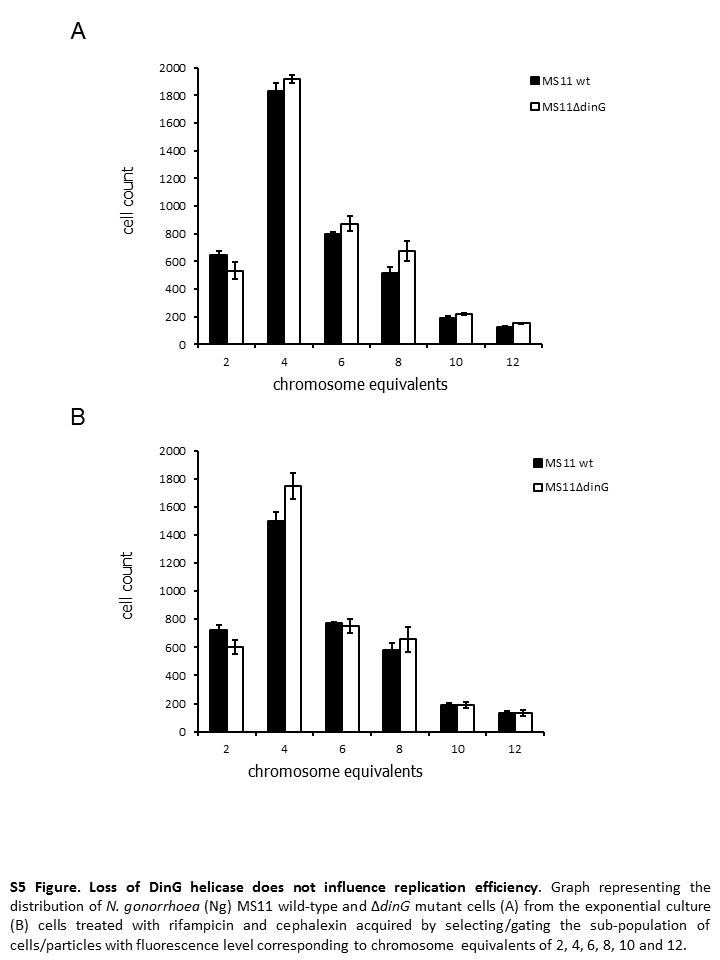

Supplement: S5 Fig — (TIF) [file pone.0187900.s005.TIF]

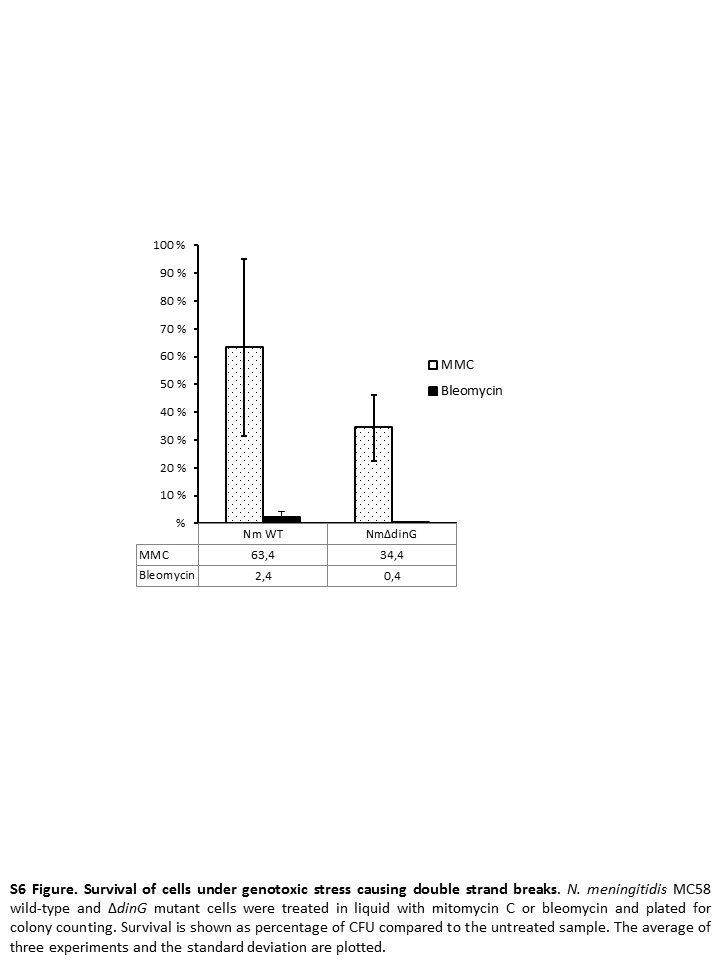

Supplement: S6 Fig — (TIF) [file pone.0187900.s006.tif]
