# Supplementary material for: The helicase DinG responds to stress due to DNA double strand breaks
Source: PLoS One. 2017 Nov 9;12(11):e0187900. doi: 10.1371/journal.pone.0187900 (PMC5679670; doi:10.1371/journal.pone.0187900)
Supplement: S2 Table — (PDF) [file pone.0187900.s008.pdf]

S2 Table: Oligonucleotides

name	sequence (5′ → 3′)	remarks	Reference
GTB5	CGTCTAGAAATAATTTTGTTTAACTTTAAGAAGGAGATATACCATGCTCACCGATTTAGAAA	*dinG, Xba*I	this study
GTB6	GCGTCGACCCCTATCCTTTTAAACGGCGGCAGGCCGGCC	*dinG, Sal*I	this study
8184OHA_AphEcoRI_REV	GCGAATTCTCATTTCGAACCCCAGAGTC	*aph, Eco*RI	this study
8186DUS_AphNheI_FOR	GCGCTAGCATGCCGTCTGAATAGACTGGGCGGTTTTATGG	*aph*, AT-DUS, *Nhe*I	this study
JEE61	ATCTTTCTAGAAGATCTCCTAC	pJet1.2	this study
JEE62	ATCTTGCTGAAAAACTCGA	pJet1.2	this study
SF177	GGCCAAAAGGTAGGCTAGCGAGGCGCCCACGCCGGTCGGCCCT	*dinG, Nhe*I	this study
SF178	CCGACCGGCGTGGGCGCCTCGCTAGCCTACCTTTTGGCCGGCGGCA	*dinG, Nhe*I	this study
SF179	CTCGAGTTTTTCAGCAAGATACCTTTGTCAGACGGCACAT	*dinG*	this study
SF180	TTCAGACGGCATGCTAGCGCCAGGTTTTTGCCGATGTTTT	*dinG*, AT-DUS, *Nhe*I	this study
SF181	GGTTCGAAATGAGAATTCGCCGTAACCATCCTCGACAACC	*dinG, Eco*RI	this study
SF182	AGGAGATCTTCTAGAAAGATCCGCTTTCCTGTTCGATAGT	*dinG*	this study
SF275	CCGCCGCCCCGGTCGAGTGAGAGCTCAATTAGCTGAGCTTGGACT	*ssb Nm* ΔC8	this study
SF276	AAGCTCAGCTAATTGAGCTCTCACTCGACCGGGGCGGCGGCA	*ssb Nm ΔC8*	this study
T1	GACGCTGCCGAATTCTACCAGTGCCTTGCTAGGACATCTTTGCCCACCTGCAGGTTCACCC		[1]
B1	ATCGATAGTCGGATCCTCTAGACAGCTCCATGTAGCAAGGCACTGGTAGAATTCGGCAGCGT		[1]
T8	TGTGGAATGCTACAGGCGTTGTAGTTTGTACTGGTGACGAAACTCAGTGTTACGGTACATGGGTT		[2]
T8-3′-3′ *	5′-TGTGGAATGCTACAGGCGTTGTAGTTTG TACTGG-3′-3′-TGACGAAACTCAGTGTTA CGGTACATGGGTT-5′		[2]
B9	GGTACCGAGCTCGAATTCACTGGCCGTCGTTCCAGTACAAACTACAACGCCTGTAGCATTCCACA		[2]
B9-5′-5′ *	3′-GTACCGAGCTCGAATTCACTGGCCGTCG TT-5′-5′-CCAGTACAAACTACAACGCCTG TAGCATTCCACA-3′		[2]

name	sequence (5′ → 3′)	remarks	Reference
EH001	TGGGATCCATGTCATTGAACAAAGTCATCCTCAT	*ssb, Bam*HI	this study
EH002	CGAAGCTTTCAGAACGGGATATCGTCGTCAATGT	*ssb, Hin*dIII	this study

* modified oligonucleotide with switched polarity

References:

1. Rossi ML, Ghosh AK, Kulikowicz T, Croteau DL, Bohr VA. Conserved helicase domain of human RecQ4 is required for strand annealing-independent DNA unwinding. DNA Repair (Amst). 2010;9(7):796-804. PubMed PMID: 20451470.
2. Voloshin ON, Vanevski F, Khil PP, Camerini-Otero RD. Characterization of the DNA damage-inducible helicase DinG from *Escherichia coli*. J Biol Chem. 2003;278(30):28284-93. PubMed PMID: 12748189.
