# Supplementary material for: The helicase DinG responds to stress due to DNA double strand breaks
Source: PLoS One. 2017 Nov 9;12(11):e0187900. doi: 10.1371/journal.pone.0187900 (PMC5679670; doi:10.1371/journal.pone.0187900)
Supplement: S1 Text — (PDF) [file pone.0187900.s009.pdf]

S1 Text

Materials and Methods

Proteomics analysis

Sample pre-treatment: Nm cells from overnight plate culture were suspended in liquid GC medium to $OD_{660} \approx 0.3$, and diluted 10 times in CO_2 saturated GC medium containing IsoVitaleX. The cells were allowed to grow for two hours at 37°C with tumbling. Then the cells were treated with MMC (10 μg/l) for 1 h. The treated and control cells were pelleted and washed three times in PBS buffer before inactivation at 60°C for 30 min. The inactivated cells were re-suspended in a lysis buffer (2% SDS/10 mM Tris-HCl, pH 7.5) containing EDTA free protease inhibitor cocktail (Roche, USA) and PhosStop (Roche, USA) and mechanically disrupted using lysing matrix B tubes in a MagNA Lyser (Roche, USA) at 6000 rpm for 90 s. To obtain a maximum protein extraction, the mechanical disruption was repeated six times with 1 min cooling on ice in between. The lysates were then cleared at 15000 × g for 15 min. The supernatant containing the total soluble protein was collected and protein concentration was determined using DirectDetect™ Spectrometer (Millipore, USA). Protein samples were aliquoted and stored at -20°C until further use.

In-solution tryptic digestion: A 100 μg soluble protein was precipitated by adding four volumes of ice-cold acetone overnight at -20 °C. Following centrifugation at 16000 × g for 15 min, precipitated proteins were vacuum dried and then solubilized in 30 μl urea buffer (6 M urea in 50 mM ammonium bicarbonate (ABC), reduced for 30 min at room temperature with 1 mM DTT and then alkylated for 15 min by 5 mM iodoacetamide. To achieve a final urea concentration below 2 M, samples were further diluted four fold in 50 mM ABC. Modified sequencing grade trypsin of porcine origin was then added at a ratio of 1:100 [w/w] (trypsin: protein ratio) and the samples were incubated overnight at 37°C with shaking at 400 rpm. The tryptic peptide mixture was then collected via centrifugation prior to desalting using a reverse phase C_{18} stop and go extraction tips protocol [1]. The samples were dried down in a vacuum drier and resuspended in 30 μl of 0.1% FA prior to liquid chromatography tandem mass spectrometry (LC-MS/MS) analysis

Mass spectrometry analysis: Samples were injected into an EASY 1000 nLC (Thermo Scientific) coupled to a Q-Exactive MS using a data-dependent Top10 method. A two-column

set up was used with a pre-column (Acclaim PepMap 100, 75 µm × 2 cm, nanoviper, C18, 3 µm, 100 Å (Thermo Scientific) and an analytical column (PepMap RSLC, C18, 2 µm, 100 Å, 50 µm × 15 cm (Thermo Scientific)). Each sample was injected in triplicates. Peptides were separated using a 120 min gradient with solvent A (0.1% FA/3% ACN (FA:LC-MS grade (Fluka), ACN: LC-MS grade (Merck)) and solvent B (0.1% FA/97% ACN) using the following steps: i) 2% to 30% B from start to 90 min, ii) 30% to 45% B from 90 min to 100 min, iii) 45% to 90% B from 100 min to 115 min, iv) 90% B from 100 min to 120 min.

Database search and statistics: MS data were analysed using MaxQuant software package version 1.4.0.5 as described by Cox and Mann [2]. MS/MS spectra were searched by the Andromeda search engine [3] against the UniProtKB FASTA database for the Nm serogroup B (strain MC58, UP000000425, UniProt) (2001 entries) using the following parameters: Enzyme specificity was set as Trypsin/P, and a maximum of two missed cleavages and a mass tolerance of 0.5 Da for fragment ion were applied. The 'requantify' and 'match between runs' options were checked with a retention time alignment window of 3 min. Oxidations (M), acetylation (protein N term), Gln-pyro (Q) and pyro-Glu (E) were specified as variable modifications and carbamidomethyl (C) as fixed modification.

Database search was performed with mass tolerance of 20 ppm for precursor ion for mass calibration, and with a 6 ppm tolerance after calibration. The maximum false peptide and protein discovery rate was specified as 0.01. Seven amino acids were required as minimum peptide length. Proteins with at least two peptides of which at least one is unique were considered as reliably identified. Following protein identification by a database search, validation for multiple comparisons was corrected using the Benjamini-Hochberg correction [4]. To aid in the control of false positives, the database was supplemented with additional sequences for common contaminants and reversed sequence of each entry. The default settings were applied for all other parameters.

The statistical determination of protein abundances was assessed using Perseus software version 1.5.1.6 [5]. Label free quantification intensity (LFQ) values were used to assess differences in the abundance of proteins between the three biological replicates of the different *Neisseria meningitidis* samples. To identify proteins whose abundance were changed significantly between samples, a combination of two tailed unpaired t test ($p \leqslant 0.05$) and fold change cut-off ±1.5 was used.

The functional categories for the significant proteins found were obtained using the KEGG and blastKOALA[6]. An in-house python script was used to retrieve and blast the sequences using blastKOALA. Proteins with existing KEGG pathway, module, or functional hierarchy (BRITE) annotations were identified.

Principal component analysis (PCA) was done using R [7].

Flow cytometry analysis

For flow cytometry, *Neisseria gonorrhoeae* (Ng) was used due to safety issues. Colonies of the wildtype strain MS11 and NgΔ*dinG* were suspended in CO_2 saturated liquid GC medium to $OD_{660} \approx 0.02$. The cell suspension was further diluted 10 fold in 10 ml liquid GC medium containing 0.5 X IsoVitaleX and cells were grown overnight at 30 rpm and 37°C to $OD_{660} \approx 0.16$. The culture was then diluted 10 fold and grown for 4 h (4 doubling time) at 37°C at 60 rpm until $OD_{660} \approx 0.14$ - 0.18. Ng has a doubling time of 60 min at 37°C [8]. A 1 ml sample from the exponentially growing cultures of non-treated cells was collected and kept on ice until further processing. To 3 ml samples, 36 µg/ml rifampicin [9] and 4 µg/ml cephalexin [10] were added and this cultures grown for an additional 6 h. The cells were fixed as described elsewhere [11, 12] and twice washed in TE buffer and collected at 18000 × g for 4 min at 4°C. The cells were resuspended in 100 µl TE buffer and fixed by addition of 900 µl 77% ethanol. The fixed cells were washed in 1 ml ice-cold 0.1 M phosphate buffer (PB) (98 mM K_2HPO_4, 2 mM KH_2PO_4, pH 9) and resuspended in 500 µl PB. The cells were protein stained with 1.5 µg/ml fluorescein isothiocyanate (FITC) overnight at 4°C and washed in 1 ml ice-cold 0.02 M Tris-buffered saline (TBS) and resuspended in 500 µl TBS. Then the cells were DNA stained with 1.5 µg/ml of Hoechst 33258 in TBS for 30 min and passed through a 5 µm Acrodisc Syringe Filter (Pall Life Sciences) to remove any aggregates. Slowly growing *E. coli* CM735, the majority having one or two chromosomes, were used as standard to calibrate the flow cytometer [12]. Sample processing was carried out on a BD LSR II flow cytometer (BD Biosciences) as previously described and the data obtained from the flow cytometer were analysed using FlowJo version 10 software [12, 13].

Survival assay

The sensitivity of *Neisseria meningitidis* to MMC and bleomycin was measured with overnight cultures grown on GC agar plates at 37°C with 5% CO_2. Cells were suspended in GC medium containing IsoVitaleX at a density giving an OD_{660} of about 0.2. MMC to a final concentration of 20 ng/ml or bleomycin to a final concentration of 10 µg/ml were added to

5 ml of the cell suspensions and the cultures were incubated in a 15 ml tube with overhead rotation of 60 rpm at 37°C for 1h.Tenfold serial dilutions were made in GC medium and 50 µl of the dilutions plated in triplicates onto plain GC agar plates. After overnight incubation at 37°C with 5% CO_2 colonies were counted. Survival was calculated as the percentage of average CFU for the treated cultures in relation to untreated cultures. The experiment was repeated three times for the Nm strains MC58 and McSAF93B (S3 Table).

References:

1. Rappsilber J, Ishihama Y, Mann M. Stop and go extraction tips for matrix-assisted laser desorption/ionization, nanoelectrospray, and LC/MS sample pretreatment in proteomics. Anal Chem. 2003;75(3):663-70. PubMed PMID: 12585499.
2. Cox J, Mann M. MaxQuant enables high peptide identification rates, individualized p.p.b.-range mass accuracies and proteome-wide protein quantification. Nat Biotechnol. 2008;26(12):1367-72. Epub 2008/11/26 09:00. PubMed PMID: 19029910.
3. Cox J, Neuhauser N, Michalski A, Scheltema RA, Olsen JV, Mann M. Andromeda: a peptide search engine integrated into the MaxQuant environment. J Proteome Res. 2011;10(4):1794-805. Epub 2011/01/25 06:00. PubMed PMID: 21254760.
4. Benjamini Y, Drai D, Elmer G, Kafkafi N, Golani I. Controlling the false discovery rate in behavior genetics research. Behav Brain Res. 2001;125(1-2):279-84. Epub 2001/10/30 10:00. PubMed PMID: 11682119.
5. Tyanova S, Temu T, Sinitcyn P, Carlson A, Hein MY, Geiger T, et al. The Perseus computational platform for comprehensive analysis of (prote)omics data. Nat Meth. 2016;13(9):731-40. doi: 10.1038/nmeth.3901.
6. Kanehisa M, Sato Y, Morishima K. BlastKOALA and GhostKOALA: KEGG Tools for Functional Characterization of Genome and Metagenome Sequences. Journal of molecular biology. 2015;14(15):006. Epub 2015/11/21 06:00. PubMed PMID: 26585406.
7. Team RC. R: A Language and Environment for Statistical Computing. 3.2.5 ed: R Foundation for Statistical Computing; 2016.
8. Tobiason DM, Seifert HS. The obligate human pathogen, Neisseria gonorrhoeae, is polyploid. PLoS Biol. 2006;4(6):e185. Epub 2006/05/25 09:00. PubMed PMID: 16719561.
9. Pagliarulo C, Salvatore P, De Vitis LR, Colicchio R, Monaco C, Tredici M, et al. Regulation and differential expression of gdhA encoding NADP-specific glutamate dehydrogenase in Neisseria meningitidis clinical isolates. Mol Microbiol. 2004;51(6):1757-72. Epub 2004/03/11 05:00. PubMed PMID: 15009900.
10. Bill NJ, Washington JA, 2nd. Comparison of in vitro activity of cephalexin, cephradine, and cefaclor. Antimicrob Agents Chemother. 1977;11(3):470-4. Epub 1977/03/01. PubMed PMID: 301005.
11. Stokke C, Flatten I, Skarstad K. An easy-to-use simulation program demonstrates variations in bacterial cell cycle parameters depending on medium and temperature. PLoS One. 2012;7(2):e30981. Epub 2012/02/22 06:00. PubMed PMID: 22348034.
12. Torheim NK, Boye E, Lobner-Olesen A, Stokke T, Skarstad K. The Escherichia coli SeqA protein destabilizes mutant DnaA204 protein. Mol Microbiol. 2000;37(3):629-38. Epub 2000/08/10 11:00. PubMed PMID: 10931356.
13. FlowJo. 10 ed: FlowJo, LLC; 2016. p. flow cytometry software.
